# Supplementary material for: Environmental DNA and visual encounter surveys for amphibian biomonitoring in aquatic environments of the Ecuadorian Amazon
Source: PeerJ. 2023 Jul 11;11:e15455. doi: 10.7717/peerj.15455 (PMC10348306; doi:10.7717/peerj.15455)
Supplement: Supplemental Information 1 [file peerj-11-15455-s001.docx]

**Supplementary Material**

**Environmental DNA and visual encounter surveys for amphibian biomonitoring in aquatic environments of the Ecuadorian Amazon**

Walter Quilumbaquin-Alba^1^, Andrea Carrera-Gonzalez ^1,2^, Christine Van der heyden^3^ and H. Mauricio Ortega-Andrade^1^

^1^ Biogeography and Spatial Ecology Research Group, Universidad Regional Amazónica Ikiam, Tena, Napo, Ecuador

^2^ Molecular Biology and Biochemistry Lab, Universidad Regional Amazónica Ikiam, Tena, Ecuador

^3^ University of Applied Sciences and Arts – HOGENT, Health and Water Technology Research Centre, Department of Biosciences and Industrial Technology, – Gent, Belgium

Email address: arman7085@email.com

**Geographical location**

**Table S1.** Geographical location of the sampling sites in the Tena River micro-basin.

| **Code** | **Location** | **Coordinates** | **Height** |
| --- | --- | --- | --- |
| ChP1 | Ponds | 0°57’39°S; 77°51’34°W | 569 m |
| ChP2 |  | 0°56’23°S; 77°51’54°W | 635 m |
| ChP3 |  | 0°56’10°S; 77°52’03°W | 649 m |
| TrP1 | River transects | 0°57’30°S; 77°51’36°W | 574 m |
| TrP2 |  | 0°56’38°S; 77°51’49°W | 615 m |
| TrP3 |  | 0°56’11°S; 77°52’06°W | 645 m |

**Protocols for DNA extraction from muscle or liver tissue samples**

The extraction was performed at the Universidad Regional Amazónica Ikiam, with the Promega commercial kit. Before the extraction, the work area was sterilized to avoid cross-contamination from previous processes. First, a solution of 120 µl of 0.5 M EDTA (pH=8) and 500 µl of Nucleic Lysis Solution (ENLS) was prepared for refrigeration, these volumes are for one sample. Between 5-10 mg of sample was used, depending on the amount of tissue available. The sample was placed in a 1.5 mL microtube and 300 µL of the previously prepared ENLS solution was added. The samples were macerated with a sterile micropestles until the sample was completely triturated. Another 300 µl of ENLS was added and the microtube was homogenized by vortexing. Next, 17.5 µl of proteinase K was added to the sample and incubated overnight at 50 °C. Before further processing, digestion was checked for completeness and the sample was allowed to stand at room temperature for 5 min. 3 µl RNase solution was added and incubated for 30 minutes at 37 °C and allowed to stand at room temperature for 5 minutes. 200 µl of Protein Precipitation Solution was added and homogenized by vortexing for 20 seconds. The samples were placed on ice for 5 minutes and centrifuged at 16 000 xg for 5 minutes, the pellet was checked for presence. The supernatant was removed to a new 1.5 mL tube and the pellet was discarded. 600 µl of cold isopropanol was added and gently homogenized by inverting the tube, centrifuged at 16 000 xg for 2 minutes and the supernatant was discarded by decanting. 600 µl of 70% ethanol was added to wash and precipitate the DNA. Centrifuged again at 16,000 xg for 2 minutes and the ethanol was discarded by decanting. All ethanol was dried, if necessary, leaving the sample overnight on a towel. Finally, the DNA was rehydrated with 50 µl of DNA rehydration solution, the sample was thoroughly homogenized and stored at 4 °C, if for a prolonged time at -80 °C.

**DNA extraction from environmental samples (water)**

The extraction of the eDNA-containing filter was performed according to Baetens [1]. The membranes obtained from the filtrate were cut into small pieces using scalpel, scissors, and forceps, previously sterilized. The small pieces of the filter were placed in a 1.5 mL microtube. Then, 300 µL of Extraction buffer was added and macerated using a micropestles for 4 minutes and 300 µL of Extraction buffer was added again. 2.5 µL of proteinase K 20 µg/ml (Promega, Madison, USA) solution was added and vortexed vigorously for 2 minutes and incubated overnight at 55 °C. The sample was cooled to room temperature for 5 min and 175 µL of MPC Protein Precipitation Reagent was added and vortexed for 20 sec. Centrifuged at 14 000 rpm for 12 min at 4 °C and placed on ice for 1 min. The supernatant was transferred to a 1.5mL microtube and 3 µL of RNAse solution (5 mg/mL) was added. It was homogenized by pipetting and the tube was inverted 25 times. It was then incubated at 37°C for 10 min. It was allowed to stand for 5 min at room temperature and centrifuged at 14 000 rpm for 6 min at 4 °C. The supernatant was transferred to a 1.5 mL microtube and placed in a cold block. 500 µL of cold isopropanol was added, the tube was inverted 30-40 times. Centrifuged at 14000 rpm at 4°C for 11 min and decanted the isopropanol and added 500 µL of 70% ethanol, the tube was inverted to wash the pellet carefully. The tube was centrifuged at 14000 rpm for 3 min and the ethanol was discarded by decanting. The obtained DNA was dried overnight by putting the inverted open tube on a clean absorbent towel. Finally, 40 µL of sterilized MilliQ was added. ADN samples were completely homogenized and stored at 4 °C, if for a prolonged time at -80 °C.

**Detailed information of the supplementary tables**

Complete information of all records performed by VES and eDNA metabarcoding is available at figshare: <https://doi.org/10.6084/m9.figshare.c.6321395.v1>

**Supplementary Tables**

**Table S2**. **List of species recorded in the Tena River Basin and their respective sequences.**

| Species | Code | Sampling year | | Sequence |
| --- | --- | --- | --- | --- |
| *Adenomera andreae* | TWQ_019 | 2020 | CGCCTCTTGCCTCAAATAAGAGGTCCAGCCTGCCCAGTGACACTGTTCAACGGCCGCGGTATCCTAACCGTGCGAAGGTAGCGTAATCACTTGTTCTTTAAATGAGGACTAGTATGAAAGGCATCACGAGAGTCATACTGTCTCCTTTTTCCAATCAGTTAAACTAATCTCCCCGTGAAGAAGCGAGGATAATTCTATAAGACGAGAAGACCCTATGGAGCTTTAAACAAATAACACTTACCTACCCCCTCCTTTAATTTCAGAAAAATCTCCCACTTTGGCATGATACTTATAAGTTTTTGATTGGGGTGATCACGGAACAAAAAACAACCTCCGCAATGAAAGACACTCCCCTAACTAAGTTAAGAACTACAGCTCTATACATCAACAAATTGACACTAATTGACCCAATATATTGATCAATGAACCAAGTTACCCTAGGGATAACAGCGCAATCTACTTCAAGAGCTCCTATCGACAAGTGGGTTTACGACCTCGATGTTGGATCAGGGTATCCTAATGGTGTAGCCGCTATTAAAGGTTCGTTTGTTCAACGATTAAAACCCTACGT | |
| *Adenomera hylaedactyla* | AY943240 | 2005 | ATAAGAGGTCTAGCCTGCCCAGTGACATTGTTCAACGGCCGCGGTATCCTAACCGTGCGAAGGTAGCGTAATCACTTGTTCTTTAAATGAGGACTAGTATGAAAGGCATCACGAGAGTCATACTGTCTCCTTTCTCTAATCAGTGAAACTAATCCCCCCGTGAAGAAGCGGGGATAGAAATATAAGACGAGAAGACCCTATGGAGCTTTAAACACATAATATATGCCCTTTTAACTTCAAATTCCAGAAAAATCTCTTATCTTGGTATAATAACTAATAGTTTTTGGTTGGGGTGACCACGGAATAAAAAACAACTTCCACAATGAAAGATTCTCCTTCACTAAGTTAAGGACTACAACCCTATACATCAATAAATTGACATAATTGACCCAACATATTGATCAATGAACCAAGTTACCCTAGGGATAACAGCGCAATCCACTTTAAGAGCTCTTATCGACAAGTGGGCTTACGACCTCGATGTTGGATCAGGGTACCCCAGTGGTGCAGCCGCTACTAAAGGTTCGTTTGTTCAACGATTAAAACCCTACGTGATCTGAGTTCAGACCGGAGTAATCCAGGTCAGTTTCTATCTATAAAGAGCTTTTTTTAGTACGAAAGGACCAAAAAAGCATGGCCAATGCTTCAATAAGCCATACCATTCATTTATGAATTTAT | |
| *Allobates aff insperatus* | TWQ_033 | 2020 | AACATCGCCTCTTGACTAACCATAAGAGGTCAAGCCTGCCCAGTGACATTGTTCAACGGCCGCGGTATCCTAACCGTGCGAAGGTAGCGTAATCACTTGTCCTTTAAATAGGGACTAGTATGAATGGCTTCACGAAAGCTATACTGTCTCCTTTATTTAATCAGTAAAACTAATTCCCCCGTGAAGAAGCGGGGATACACCTATAAGACGAGAAGACCCTGTGGAGCTTTAAACACTTTTTAAACATTTGAATTTACACTCTTATTCTATCTCTACTAAATCACTCATTTTGTTTTAAGTTTTAGGTTGGGGTGACCACGGAGAATAAATTAACCTCCACGTAGAAAGAACCTTATTTTCTAAGCAAAAAATCACATTTTTATGCATCAAAATATTTGACCTAAATTGACCCAATTTTTTGATCAACGAACCAAGTTACCCTAGGGATAACAGCGCAATCCGCTTTAAGAGCTCCTATCGACAAGCGGGCTTACGACCTCGATGTTGGATCAGGGTA | |
| *Allobates femoralis* | KX044277 | 2016 | ACATCGCCTCTTGATCAATTATAAGAGGTCAAGCCTGCCCAGTGACATTGTTCAACGGCCGCGGTATCCTAACCGTGCGAAGGTAGCGTAATCACTTGTCCTTTAAATAAGGACTAGTATGAACGGCTTCACGAAGGCTATACTGTCTCCTTAATCTAATCAATTAAACTAATCTCCCCGTGAAGAAGCGGGGATTCGTCTATAAGACGAGAAGACCCTATGGAGCTTTAAACATCTAAGACACTTGATTTTACCCACTAAACTTCATGTAACCTATCATTTTGTCTTAAGTTTTAGGTTGGGGTGACCACGGAGAAAAATTAAACCTCCACGTAGAAAGAAAATTCTTTTCTAAGCAAAGAGCCACATCTTAACGCATCAACATATTGACTTAAATTGACCCAATTTCTTGCTCAACGAACCAAGTTACCCTAGGGATAACAGCGCAATCCGCTTTAAGAGCTCCTATCGACAAGCGGGCTTACGACCTCGATGT | |
| *Allobates zaparo* | AY263227 | 2003 | AGGTCAAGCCTGCCCAGTGACATTGTTCAACGGCCGCGGTATCCTAACCGTGCGAAGGTAGCGTAATCACTTGTCCTTTAAATAAGGACTAGTATGAACGGCTTCACGAAGGCTATTCTGTCTCCCTTATCTAATCAGTTAAACTAATCTCCCCGTGAAGAAGCGGGGATTAACCTATAAGACGAGAAGACCCTATGGAGCTTTAAATATCTAAGACACTTGATTTTATTATTAAACTTCATGTAACCTATCATTTATGCCTTAAGTTTTAGGTTGGGGCGACCGCGGAGAAAAATCGAACCTCCACGTAGAAAGAAAATTTTCTTCTAAGCAAAAAGACACATCTTAACGCATCAATATATTGACCTAAATTGACCCAATTTCTTGCTCAACGAACCAAGTTACCCTAGGGATAACAGCGCAATCCGCTTTGAGAGCTCCTATCGACAAGCGGGCTTACGACCTCGATGTTGGATCAGGGTATCCTAATGGTGCAGCAGCTATTAACGGTTCGTTTGTTCAACGATTAAAAC | |
| *Ameerega bilinguis* | DQ502225 | 2006 | AACATCGCCTCTTGTCTAATTATAAGAGGTCCAGCCTGCCCAGTGACTTTGTTCAACGGCCGCGGTATCCTAACCGTGCGAAGGTAGCGTAATCACTTGTTCTTTAAATGAGGACTAGTATGAATGGCCCCACGAGGGCTGCACTGTCTCCTTTTTCTAATCAATGAAACTAATCTCCCCGTGAAGAAGCGGGGATAATACTATAAGACGAGAAGACCCTATGGAGCTTTAAACAACTGAAACATTTGCTTTTCTATCAACCTCTTCCGAGCTCTTTATATTACTTAAGCATTTTTACTTCTAGTTTTAGGTTGGGGTGACCACGGAGCAAAATTAAACCTCCATGAAGAAATGAATACTTTTAAGCCACAAACTACTTTTTTAAGCATCAACAAATTGACCTTCATTGACCCAATATATTGATCAACGAACCAAGTTACCCTAGGGATAACAGCGCAATCTACTTCAAGAGCTCATATCGACAAGTAGGTTTACGACCTCGATGTTGGATCAGGGTATCCTAGTGGTGCAGCCGCTACTAATGGTTCGTTTGTTCAACGATTAAAACCCT | |
| *Ameerega parvula* | AF124125 | 2000 | GTCCAGCCTGCCCAGTGACTTTGTTCAACGGCCGCGGTATCCTAACCGTGCGAAGGTAGCGTAATCACTTGTTCTTTAAATGAGGACTAGTATGAATGGCCCCACGAGGGCTGCACTGTCTCCTTTTTCTAATCAATGAAACTAATCTCCCCGTGAAGAGGCGGGGATAGCCCTATAAGACGAGAAGACCCTATGGAGCTTTAAACAACTGAGACACTTGCTTTTCTTTTAATCTCTCCAGAGCTCTTTATTTTACTTAAGCATTTTTATCTCTAGTTTTAGGTTGGGGTGACCACGGAGCAAAATTAAACCTCCATGAAGAAATGACTTCTTTTTAAGCCACAAACTACTCTTTTAAGCATCAACAAATTGACCTTCATTGACCCAATATATTGATCAACGAACCAAGTTACCCTAGGGATAACAGCGCAATCTACTTCAAGAGCTCATATCGACAAGTAGGTTTACGACCTCGATGTTGGATCAGGGTATCCTAGTGGTGCAGCCGCTACTAATGGTTCGTTTGTTCAACGATTAAAACCCTACGTGATCTGAGT | |
| *Boana appendiculata* |  |  | No sequence record at NCBI for mitochondrial gene 16S | |
| *Boana boans* | KP148456 | 2014 | CGCCTCTTGAATAAAATAAGAGGTCCAGCCTGCCCAGTGAAATTATTCAACGGCCGCGGTATCCTAACCGTGCGAAGGTAGCGTAATCACTTGTTCTTTAAATGAGGACTAGTATGAATGGCACCACGAGGGTTCTACTGTCTCCTTTTTTTAATCAGTGAAACTAATCTCCCCGTGAAGAAGCGGGGATCTTTATATAAGACGAGAAGACCCTATGGAGCTTTAAACAACCAACATTTGCTACACCATAACATTTTTCCCTAAAAGAAGTATAGCATTATGATTATTAGTTTTCGATTGGGGTGATCACGGAGTAAAATATAACCTCCATGCCGAAAGGGTTTTACCCTGAGCAAAAAGCCACAGCTTTAAGCACTAAAAAATTAACGCAGTTTGACCCAATATTTTGATCAACGAACCAAGTTACCCTAGGGATAACAGCGCAATCCACTTCAAGAGCTCCTATCGACAAGTGGGTTTACGACCTCGATGTTGGATCAGGATATCCAAGTGGTGCAGCCGCTACTAATGGTTCGTTTGTTCAACGATTAAAACCCTA | |
| *Boana geographica* | TWQ_009 | 2020 | AACATCGCCTCTTGAGTAAAATAAGAGGTCCAGCCTGCCCAGTGAAATTATTTAACGGCCGCGGTATCCTAACCGTGCGAAGGTAGCGTAATCACTTGTTCTTTAAATGAGGACTAGTATGAATGGCACCACGAGGGTTCTACTGTCTCCTTTTTTAAATCAGTGAAACTAATCTCCCCGTGAAGAAGCGGGGATTTTTTTATAAGACGAGAAGACCCTATGGAGCTTTAAACAAACATCATTTGCTGCACATAACACTTTTACCTTTAATAAATATAGCATTATGACCTTTAGTTTTTGATTGGGGTGATCACGGAGTAAAATGAAACCTCCACGCCGAAAGGGTTAACACCCTGAGCAAAAAGCCACAACTTTAAGCACTAAAAAATTAACGTAACTTGACCCAATATTTTGATCAACGAACCAAGTTACCCTAGGGATAACAGCGCAATCCACTTCAAGAGCTCCTATCGACAAGTGGGTTTACGACCTCGATGTTGGATCAGGGTATCCTAGTGGTGCAGCCGCTACTAATGGTTCGTTTGTTCAACGATTAAAACCCTACGTG | |
| *Boana lanciformis* | TWQ_1743 | 2020 | AAAAACATCGCCTCTTGACAAAATATAAGAGGTCCAGCCTGCCCAGTGACCTAGTTCAACGGCCGCGGTATCCTAACCGTGCGAAGGTAGCGTAATCACTTGTTCTTTAAATGAGGACTAGTATGAATGGCACCACGAGGGTTAAGCTGTCTCCTTTTTCTAATCAGTGAAACTAATCTCCCCGTGAAGAAGCGGGGATTATATTATAAGACGAGAAGACCCTATGGAGCTTAAAACAAATTAACACTTGCTTCTGCCTGATTCACTTCATGAACTGACACATAAAGTATAGCACCACTGATTAATAGTTTTCGATTGGGGTGATCATGGAGTAAAAAATAACCTCCACGACGAAAAGGGGCACCTCCCTGAGCCAAAAGCCACAACTTTAAGCACTAAAAAATTGACGCACCTTGACCCAAATTTTTGAGCAACGAACCAAGTTACCCTAGGGATAACAGCGCAATCCACTTCAAGAGCCCCTATCGACAAGTGGGTTTACGACCTCGATGTTGGATCAGGGTGTCCCAGTGGTGCAGCCGCTACTAAAGGTTCGTTTGTTCAACGATTAAAACCCTACGTGA | |
| *Boana punctata* | TWQ_0710 | 2020 | CGCCTCTTGACTAGTTATAAGAGGTATAGCCTGCCCAGTGACACATGTTTAACGGCCGCGGTATCCTAACCGTGCAAAGGTAGCGTAATCACTTGTTCTTTAAATGAGGACTAGTATGAACGGCACCACGAAGGTTATACTGTCTCCTTTCTTTAATCAGTAAAACTAATCTCCCCGTGAAGAAGCGAGGATAAATTTATAAGACGAGAAGACCCTATGGAGCTTTAAACTAACCAGCATTTGCTTTATATAAATTTTTAGCAACTTTAAAGACATCTAGCCCTCTGACTGGTAGTTTTCGATTGGGGTGATCACGGAACAAAAAATAACCTCCGTGTCGAAAGGATATACCTGAGCAAAAAGCGACAGCTTTCCGCACTAAAAAATTAACGTTCATTGACCCAATATTTTGATCAACGAACCAAGTTACCCTAGGGATAACAGCGCAATCTACTTCAAGAGTCCCTATCGACAAGTAGGTTTACGACCTCGATGTTGGATCAGGGTATCCCAGTGGTGCAGCCGCTACTAAAGGTTCGTTTGTTCAACGATTAAAACCCTA | |
| *Bolitoglossa peruviana* | MG944406 | 2013 | AAACATCGCCTTTTGAAATATATACATAAAAGGTCCTGCCTGCCCAATGACTTGTTTTAATGGCCGCGGTATTATGACCGTGCAAAGGTAGCGTAATCATTTGTCTTCTAAATAAAGACCAGTATGAACGGCAAGACGAAAGCCTCACTGTCTCCCTAACCCAATCAATGAAATTGATCTTCCCGTGCAGAAGCGGGAATAATAATATAAGACGAGAAGACCCTGCGGAGCTTTAAACCCTGATTAATAATTATTATTATCTTAATACCAAGTTTTAGGTTGGGGCGACCATGAGGCACAACATAACCCTCACGATATATACCAAGAAGGACACTTCAAATAACTAACACTAGCACTTTGACCCAATAATTGACCAACGAACCAAGTTACCCCAGGGATAACAGCGCAATCCGCTCAAAAAGTCCTTATTAACGAGCAGGTTTACGACCTCGATGTTGGATCAGGACACCCAAATAGTGCAACAGCCATTAAAGGTTCGTTTGTTCAACGATTAAAGTCCTACGTGATCTGAGTTCCAGACCGGA | |
| *Caecilia dunni* |  |  | No sequence record at NCBI for mitochondrial gene 16S | |
| *Cochranella resplendens* | TWQ_035 | 2020 | AACATCGCCTCTTGCCTAAATATAAGAGGTCCAGCCTGCCCAGTGACTCTGTTCAACGGCCGCGGTATCCTAACCGTGCGAAGGTAGCGTAATCACTTGTTCTTTAAATGAGGACTGGTATGAATGGCACCACGAAGGTTATACTGTCTCCTCTTTTTAATCAGTGAAACTAATCTCCCCGTGAAGAAGCGGGGATAAACATATAAGACGAGAAGACCCTATGGAGCTTTAAACTATGTAACACTTGTACTAACCCTAATACAAATTCCAGAAAAATCCTCTACATCTATACAAGATTGTTGCCAGTTTTAGGTTGGGGTGACCACGGAGCAAAAAACAACCTCCACATTGAAAGGAGTTTATTCCTTAACCAAGAGCCACACCTCCACGTATCATTACAATGACATTAATTGACCCAATATATTGATCAACGAACCAAGTTACCCTAGGGATAACAGCGCAATCCACTTCAAGAGCTCCTATCGACAAGTGGGTTTACGACCTCGATGTTGGATCAGGGTATCCCAGTGGTGCAGCCGCTACTAAAGGTTCGTTTGTTCAACGATTAAAACCCTAC | |
| *Dendropsophus bifurcus* | TWQ_1113 | 2020 | AAAAACATCGCCTCTTGCACAATATAAGAGGTCCAGCCTGCCCAGTGACTTAGTTCAACGGCCGCGGTATCCTAACCGTGCGAAGGTAGCGTAATCACTTGTTCTTTAAATGAGGACTAGTATGAATGGCACCACGAGGGTTACACTGTCTCCTTTTTCCAATCAATAAAACTGATCTTCCCGTGAAGAAGCGGGAATTATAATATTAGACGAGAAGACCCTATGGAGCTTCAAACTATATAGCAYTGACTTTTACACTTTACTCTTCWGAGTCCTACACTTATTCTGCTCTTTTTGACTATTAGTTTTGGGTTGGGGTGACCGCGGAGCAAAGATTAACCTCCATGATGAACAAAACCGCTTTTGAACCATAAGCCACAACTTTAAGCACCAATAAATTGACATAAATTGACCCATTTTTTGACCAACGAACCAAGTTACCCTAGGGATAACAGCGCAATCTACTTCAAGAGCCCCTATCGACAAGTGGGTTTACGACCTCGATGTTGGATCAGGGTATCCTAGTGGTGCAGCCGCTACTAAAGGTTCGTTTGTTCAACGATTAAAACCCTACGTGATCT | |
| *Dendropsophus marmoratus* | MT503915 | 2020 | AAAAACATCGCCTCTTGAAACTTATAAGAGGTCCAGCCTGCCCAGTGACTAAGTTCAACGGCCGCGGTATCCTAACCGTGCGAAGGTAGCGTAATCACTTGTTCTTTAAATGAGGACTAGTATGAATGGCACCACGAGGGTGGTACTGTCTCCTTTCTCTAATCAATGAAACTGATCTCCCCGTGAAGAAGCGGGGATGAAAATATAAGACGAGAAGACCCTATGGAGCTTTAAATCATAGAGCACTGACTTACTAATAACAAAGTTTCAGAACACTAATAACCAGTCTAGTCAGAATGACTATAGATTTTAGGTTGGGGTGACCGCGGAGAAAAAATTATCCTCCACATTGAACGGAGCTACTCCTGAGCAGAAAGCTACAACTTTAAGCACCAACAAATTGACATTAATTGACCCAATATATTGATCAACGAACCAAGTTACCCTAGGGATAACAGCGCAATCTATCTCAAGAGCCCCTATCGACAGGTAGGTTTACGACCTCGATGTTGGATCAGGGCATCCTAGTGGTGCAGCCGCTACTAAAGGTTCGTTTGTTCAACGATTAAAGCCCT | |
| *Dendropsophus parviceps* | TWQ_0304 | 2020 | TTGCCTAAATATAAGAGGTCTAGCCTGCCCAGTGACTTAGTTCAACGGCCGCGGTATCCTAACCGTGCGAAGGTAGCGTAATCACTTGTTCTTTAAATGGGGACTAGTATGAATGGCATCACGAGGGTTATGCTGTCTCCTTTTTCTAATCAATGAAACTGATCTCCCCGTGAAGAAGCGGGCATAATAATATAAGACGAGAAGACCCTATGGAGCTTTAAACGATTATAGCACTGGCCCACTCATTCCATGTTTCAGAACTACAACATTCACTATAGCCTTAATGACTATTAGTTTTAGGTTGGGGTGACCGCGGAGCAAAAATTAACCTCCACATTGRAAAGAACCTTTCTTGAGCCCAAAGCCACAACTTCAAGCACCAATAAACTGACATACATTGACCCAAGAAATTGATCAACGAACCAAGTTACCCTAGGGATAACAGCGCAATCCACTTCAAGAGCCCCTATCGACAAGTGGGTTTACGACCTCGATGTTGGATCAGGGTATCCCAGTGGCGCAGCCGCTACTAACGGTTCGTTTGTTCAACGATTAAAACCCT | |
| *Dendropsophus reticulatus* | TWQ_2131 | 2020 | AAAAACATCGCCTCTTGCATGATATAAGAGGTCTAGCCTGCCCAGTGACCCAGTTCAACGGCCGCGGTATCCTAACCGTGCGAAGGTAGCGTAATCACTTGTTCTTTAAATGAGGACTAGTATGAATGGCATCACGAGGGTTACACTGTCTCCTTTTTCTAATCAATGAAACTAATCTCCCCGTGAAGAAGCGGGGATATGTACATAAGACGAGAAGACCCTATGGAGCTTCAAACTATTTAGCACTGGCTTTTATATATCCCTACTTCAGAGTTTTTACACTTATTCTAGCCTCTTGACTAATAGTTTTAGGTTGGGGTGACCACGGAGCAAAAATTAACCTCCACGATGAACAGAGCTACTCTTGAACCAAAAGCCACAACTTTAAGCACCAATAAACTGACACAAATTGACCCAATATTTTGAACAACGAACCAAGTTACCCTAGGGATAACAGCGCAATCCACCTTAAGAGCCCCTATCGACAGGTGGGTTTACGACCTCGATGTTGGATCAGGATATCCCAGTGGTGCAGCCGCTACTAATGGTTCGTTTGTTCAACGATTAAAACCCTACGTGATCTG | |
| *Dendropsophus rhodopeplus* | TWQ_0625 | 2020 | ATCGCCTCTTGACCCTTTATAAGAGGTCCAGCCTGCCCAGTGACTAAGTTCAACGGCCGCGGTATCCTAACCGTGCGAAGGTAGCGTAATCACTTGTTCTTTAAATAAGGACTAGTATGAATGGCACCACGAGGGTTATACTGTCTCCTTTTTCTAATCAATGAAACTGATCTCCCCGTGAAGAAGCGGGGATAAATATATAAGACGAGAAGACCCTATGGAGCTTCAAATGACATAGCACTGGCCCTTTCACTTCACATTTCAAAAACACTATATTTAAACAAGCCAAAATGACTATTTATTTTAGGTTGGGGTGACCACGGAGCAAAAACTAACCTCCATGCTGAACGGAGCTACTCCTTAGCTAAAAGCTACAGCTTTAAGCAACAACAAACTGACACCAATTGACCCAATACTTTGATCAACGAACCAAGTTACCCTAGGGATAACAGCGCAATCCATTTCAAGAGCTCCTATCGACAAGTGGGTTTACGACCTCGATGTTGGATCAGGGTATCCCAGTGGTGCAGCCGCTACTAAAGGTTCGTTTGTTCAACGATTAAAACCCT | |
| *Dendropsophus sarayacuensis* | TWQ_0102 | 2020 | TCGCCTCTTGAGCCCTATAAGAGGTCTAGCCTGCCCAGTGACTTAGTTTAACGGCCGCGGTATCCTAACCGTGCGAAGGTAGCGTAATCACTTGTTCTTTAAATGAGGACTAGTATGAATGGCATCACGAGGGTTCCACTGTCTCCTTTTTCCAATCAATGAAACTGATCTTCCCGTGAAGAAGCGGGAATCTTAATATTAGACGAGAAGACCCTATGGAGCTTCAAACTAAATAGCACTGGCTTTTCCCCTATATACCTCAAGGGGTATTAACCCTTATTCTAGCCTTTTAACTATTAGTTTTAGGTTGGGGTGACCGCGGAGCAAAAATTAACCTCCACAATGAATAGCACTATTCTTGAGCCAAAAGCCACAACTTTAAGCACTAACAAACTAACATAAATTGACCCAATATTTTGAACAACGAACCAAGTTACCCTAGGGATAACAGCGCAATCCTCTTCAAGAGCCCTTATCGACAAGTGGGTTTACGACCTCGATGTTGGATCAGGATATCCCAGTGGTGCAGCCGCTACTAAAGGTTCGTTTGTTCAACGATTAAAATCCTACGT | |
| *Dendropsophus triangulum* | MT503958 | 2020 | AAAAACATCGCCTCTTGCACCAATATAAGAGGTCCAGCCTGCCCAGTGACCCAGTTCAACGGCCGCGGTATCCTAACCGTGCGAAGGTAGCGTAATCACTTGTTCTTTAAATGAGGACTAGTATGAATGGCACCACGAAGGTTACACTGTCTCCTTTTTCTAATCAATGAAACTAATCTCCCCGTGAAGAAGCGGGGATTTAAATATAAGACGAGAAGACCCTATGGAGCTTCAAACTAAATAGTATTGGCTTTTATCCATTTACACCTCCGGGGCCTCATACTAACTCTAGCCTTTTAACTATTAGTTTTGGGTTGGGGTGACCGCGGAGCAAAAATTATCCTCCATGATGAATAGAGCTACTCTTGAGCCAAAAGCCACAACTTTAGGCACCAATAAACTGACATAAATTGACCCAATATTTTGAGCAACGAACCAAGTTACCCTAGGGATAACAGCGCAATCCATCTCAAGAGCCCCTATCGACAGGTGGGTTTACGACCTCGATGTTGGATCAGGGTATCCCAGTGGTGCAGCCGCTACTRAAGGTTCGTTTGTTCAACGATTAAAACCCT | |
| *Engystomops petersi* | KY814625 | 2017 | TCTTGCTTACNCCNNGAAAATGTATGTGAAAACCATACCGTTTTGAGCTAAAAACTTAGCCTTACCCCTACAATTATATTTACCCTATTTTACTTACACAAAATAAAACATTCTTTAAGCTTAGTAAAGGAGATAAAAAAGTTTTTTAGAGCTATAAAATAAGTACCGCAAGGGAAAAATGAAATAAAAATGAAACAAAATTCAAGCATAATATAGTAGAGTTTTCCCCTCGTACCTTTTGCATCATGATTCAACTAGTTTAACCACGCAAAAAGAATTTAAGTTTGACTTCCCGAAACTAAGTGAGCTACTTCAGGGCAGCTAAATGAGCCAACCCGTCTCTGTTGCAAAAGAGTGGGATGACCCTTAAGTAGAGGTGATAAACCTAACGAACTTAGTGATAGCTGGTTATTCAGGAAAAGAATTTTAGTTCAACTTAAAATTACATAAATAAAATAAAATAAAATTTTAATTTTAAAGCAATTCAAATAAGGTACAGCCTATTTGAAATAGAATACAATCTAAACTATAGGGTAAATATAAAAAATTAATGTAATAAAGTAGGCCTAAAAGCAGCCAACTTTAAAATAGCGTCAAAGCTTACTTCCATAATTTTTTAATACTATTATTTACAATGAACCCTTTATTTTTACTGAATATTTCTATAAAGTTATAGAACCTTTTATGTTGAAACTAGTAACAAGAAGTTAAACTTCTCCTCAATATAAGTATAAGTCGACTAGGATAAACCATCGACAATTAACGCTTAAATTAAATGTATTAACTCCACAAGAAAAACTTACATTACCCCACGTTNNTCTTACACCAG | |
| *Gastrotheca testudinea* | KR270430 | 2015 | CGCCTCTTGAGAAGCATAAGAGGTCCAGCCTGCCCAGTGACAATGTTCAACGGCCGCGGTATCCTAACCGTGCGAAGGTAGCGTAATCACTTGTCCTTTAAATGAGGACTAGTATGAATGGCACCACGAGGATTATACTGTCTCCTTTTTCCAATCAGTGAAACTAATCTTTCCGTGAAGAAGCGGAAATACATCTATAAGACGAGAAGACCCTATGGAGCTTTAAACGAAACATCATTTGCTATAGCACACACTAATCCCAGGAAATCAACCTTTATTTTAGCATTATAATTGTTAGTTTTAGGTTGGGGTGACCGCGGAGTAAAAAATAACCTCCACAATGAACTGGGGAACTCCCCTAAACAAAAAACCACAACTTTATGTATCAATAAATTGACATCAATTGACCCAATATTTTGATCAACGAAACAAGTTACCCTAGGGATAACAGCGCAATCCACTTTGAGAGCCCATATCGACAAGTGGGTTTACGACCTCGATGTTGGATCAGGGTACCCCAGTGGTGCAGCCGCTACTAAAG | |
| *Hemiphractus bubalus* | DQ679412 | 2006 | AAAACATCGCCTCTTGTCTAAACCATAAGAGGTCCAGCCTGCCCAGTGATACTATTCAACGGCCGCGGTATTTTAACCGTGCAAAGGTAGCGCAATCACTTGTCCTTTAAATGCGGACTAGTATGAACGGCACCACGAAAGTTAAACTGTCTCCTTTTTCTAATCAGTGAAACTAATCTCCCCGTGAAAAAGCAGGGATAAATATATAAGACGAGAAGACCCTATGGAGCTTTAAATATTAACAACAATTGCCACAACACCTATGATTGTTAATTTTAGGTTGGGGCGACCACGGAGTAAAACCCAGCCTCCATGCCAAATATAGCAAAAATCTACAACTTTAAGCATCATTAAATTGACACTTATTGACCCAATATTTTGATCAACGAACCAAGTTACCCTAGGGATAACAGCGCCATCCACTTCAAGAGCCCATATCGACAAGTGGGCTTACGACCTCGATGTTGGATCAAGGTACCCCAGTGGCGCAGCCGCTACTAAAGGTTCGTTTGTTCAACGATTAA | |
| *Hemiphractus scutatus* | MG011479 | 2018 | GGTCCAGCCTGCCCAGTGACTTTATTCAACGGCCGCGGTATCCTAACCGTGCAAAGGTAGCGTAATCACTTGCCCTTTAAATAAGGACTAGTATGAACGGCATCACGAAGGTTAAACTGTCTCCCCTTTTTAATCAGTGAAACTATCCCCCCCGTGAAGAGTCGGGGATATATCTACAAGACGAGAAGACCCTATGGAGCTTTAAATTCTAACAACAATTGCTCTTAAATATATTTAGCATTTATGATTGTTAATTTTAGGTTGGGGCGACCACGGAGAAAAACCTAACCTCCATGCTGACATAGTAAAACTACAATTTTAATACCATTAAATTGGCATCCATTGACCCAATATTTTGATCAACGAACCAAGTTACCCTAGGGATAACAGCGCCATCCACTTCAAGAGCTCATATCGACAAGTGGGTTTACGACCTCGATGTTGGATCAGGGTATCCCAGTGGTGCAGCCGCTACTAAA | |
| *Hyalinobatrachium munozorum* | JF266570 | 2011 | AATATAGAAGATATTATGTTAAAACTAGTAACAAGAAGAAGAACTTCTCCTACAATGCAAGTGTAAATCAGACAGGACACCCCACTGATAATTAACACCAATGAACATAAAATAGCAACCCAACAAGAAAACCCTACTACACCATATGTTAAACTGACACCAGAGCATTTCAAGAAAGATTTAAAGAAAAAGAAGGAACTCGGCAAACATTAACCTCGCCTGTTTACCAAAAACATCGCCTCTTGAATAAGTATAAGAGGTCCAGCCTGCCCAGTGACAATGTTCAACGGCCGCGGTATCCTAACCGTGCGAAGGTAGCGTAATCACTTGTTCTTTAAATAAGGACTGGTATGAACGGCACCACGAAGGTTATACTGTCTCCTCTTTCTAATCAGTGAAACTAATCTCCCCGTGAAGAAGCGGGGATAAGAATATAAGACGAGAAGACCCTATGGAGCTTTAAACTACAAGGCACTTGCCCCACATAACCGAATTCCAGAAAATCACTACCACCCAGGCAATATTGACCCTTAGTTTTAGGTTGGGGTGACCGCGGAGCAAAAAAGAACCTCCACATTGAAAGGAGAACACTCCTTAGCCAAGAGCCACACCTCTAAGCACCAACAAAAAGACATTAATTGACCCAATATTTTGATCAACAACCAAGTTACCCTAGGGATAACAGCGCAATCCACTTCAAGAGCTCCTATCGACAAGTGGGTTTACGACCTCGATGTTGGATCAGGGTACCCCGGTGGTGCAGCCGCTACCAAAGGTTCGTTT | |
| *Hyloscitus albopunctulatus* |  |  | No sequence record at NCBI for mitochondrial gene 16S | |
| *Hyloscirtus phyllognathus* | KT279545 | 2015 | CACATAAACCCCTTTATTACCACTAGACAATTCTATATAACTATAGAAATTATTTTGTTAAAACTAGTAACAAGAAATAAACCTTCTCTTTAATATAAGTATATGCCAGAAAGGATAAACCACTGGCCATTAACATACTTGAAAATTAAGCAGCAACACCACAAGAGAACCCTGCCTATATGTATGTTAATTTTACACTAAAACATTTCAAGAAAGATTAAAAGAAAAAGAAGGAACTCGGCAAACATAAATCTCGCCTGTTTACCAAAAACATCGCCTCTTGAAACCTATATAAGAGGTCACGCCTGCCCGGTGACTCATGTTTAACGGCCGCGGTATCCTAACCGTGCGAAGGTAGCGTAATCACTTGTTCTTTAAATAAGGACTAGTATGAATGGCCACACGAAGATTTTACTGTCTCCTTTTTCTAATCAGTGAAACTAATCTCCCCGTGAAGAAGCGGGGATAAAAATATAAGACGAGAAGACCCTATGGAGCTTTAAACACAGCAGCACTTGTTAACCTTCAAACTTTCAAATAAAACTTATACATCATGACTGCAAGTTTTAGGTTGGGGTGACCACGGAGCAAAAAATAACCTCCATGTAGAACAGATTTTTCCATGAGCAAAAAACCTCAACTTTATGCACCAACAAATTGACTTCTATTGACCCAATATTTTGATCAACGAACCAAGTTACCCTAGGGATAACAGCGCAATCCACTTTAAGAGCCCCTATCGACAAGTGGGCTTACGACCTCGATGTTGGATCAGGGTACCCTAGCGGTGTAGCCGCTGCTAATGGTTCGTTTGTTCAACGAT | |
| *Hyloxalus yasuni* | MF624236 | 2017 | AACATCGCCTCCTGATACTCCCATAGGAGGTCCAGCCTGCCCAGTGACATTGTTCAACGGCCGCGGTATCCTAACCGTGCGAAGGTAGCGTAATCACTTGTTCTTTAAATGAGGACTAGTATGAACGGCTTCACGAGGGCTATACTGTCTCCTCTTTCTAATCAATGAAACTAATCTCCTCGTGAAGAAGCGAGGATAAACCTATAAGACGAGAAGACCCTATGGAGCTTTAAACAACTGAAATACTTGCTTACAACTCTTAACTTCCCGAAAACACTATTCAACTAAGCACTTTATTCATAGTTTTAGGTTGGGGTGACCACGGAGCATAAATCCACCTCCATGCTGAAAGAAGGTTCTCTTCTTATCAAAGAGCTTCAGCTCAATGCATTAATAAATTGACTCCCATTGACCCAATATTTTGATCAACGAACCAAGTTACCCTAGGGATAACAGCGCAATCTATTTCAAGAGCTCATATCGACAAATAGGTTTACGACCTCGATGTTGGATCAGGGTATCCTAGTGGTGCAGCCGCTACTAACGGTTCGTTTGTTCAACGATTAAAACCCT | |
| *Leptodactylus knudseni* | KP149492 | 2011 | CGCCTCTTGCATACCTATAAGAGGTCCAGCCTGCCCAGTGACTCTGTTCAACGGCCGCGGTATCCTAACCGTGCGAAGGTAGCGTAATCACTTGTTCTTTAAATGAGGACTAGTATGAATGGCATCACGAAGGTTATTCTGTCTCCTCTTTCCAATCAGTGAAACTAATCTCCCCGTGAAGAAGCGGGGATAACTCTATAAGACGAGAAGACCCTATGGAGCTTTAAACTAAGAATCAACTGACTTCCCACTTTTAAATTCCAGAAAACTAACTTTTACCAAGACACTCTGATTTCTAGTTTTAGGTTGGGGTGACCACGGAGTAAAAACTAACCTCCGCAATGAACGGGGATCTTCCCTTAACCAAGGACCACAACCCTAAGAATCAACACATTGACATCAATTGACCCAAAAATTTGATCAATGAACCAAGTTACCCTAGGGATAACAGCGCAATCCACTTTAAGAGCCCATATCGACAAGTGGGTTTACGACCTCGATGTTGGATCAGGGTATCCCAGTGGTGCAGCCGCTACTAATGGTTCGTTTGTTCAACGATTAAAACCCTA | |
| *Leptodactylus wagneri* | TWQ_2940 | 2020 | GGAACCCATAAGAGGTCCAGCCTGCCCAGTGACCCTGTTCAACGGCCGCGGTATCCTAACCGTGCGAAGGTAGCGTAATCACTTGTTCTTTAAATGAGGACTAGTATGAATGGCACCACGAAGGTTATACTGTCTCCTTCCTCTAATCAGTGAAACTAATCTCCCCGTGAAGAAGCGGGGATAAGCCTATAAGACGAGAAGACCCTATGGAGCTTTAAACACAGTAATAAATGCCCCCCCCCCTTTTTCTATTCAAATCTCCGGAAAACTACTTTATCTGAGCATCCTATTTACAAGTTTTAGGTTGGGGTGACCGCGGAGTAAAAAATAACCTCCACAGTGAATGGAATCTTTTTCCTAAACTCAGGGCCACAACCCTAAAAATCAATAAATTGACACCCATTGACCCAATATTTTGATCAATGAACCAAGTTACCCTAGGGATAACAGCGCAATCCACTTCAAGAGCTCCTATCGACAAGTGGGTTTACGACCTCGATGTTGGATCAGGGTACCCCAGTGGTGCAGCCGCTGCTAAAGGTTCGT | |
| *Lithodytes lineatus* | AY326012 | 2004 | ATAAGAGGTCCAGCCTGCCCAGTGACTCTGTTCAACGGCCGCGGTATCCTAACCGTGCGAAGGTAGCGTAATCACTTGTTCTTTAAATAAGGACTAGTATGAAAGGCACCACGAGGGTTGCACTGTCTCCTTTCTCTAATCAGTGAAACTAATCTTCCCGTGAAGAAGCGGGAATAAAAATATAAGACGAGAAGACCCTATGGAGCTTTAAACTAAATAATAATAGCCTACTCATTTACACAACTCCAGATGAATACTTTACCCTGGCTCGATAATTATTAGTTTTTGGTTGGGGTGACCACGGAGAAAAAAGAAACCTCCGCAATGAAAGACTCTCCTTCTTAGTTTAGGACTACTTTCCTACGCATCAATAAATTGACACATATTGACCCAACAACGTTGATCAATGAACCAAGTTACCCTAGGGATAACAGCGCAATCCACTTCAAGAGCCCCTATCGACAAGTGGGTTTACGACCTCGATGTTGGATCAGGGTATCCCAGTGGTGCAGCCGCTACTAAAGGTTCGTTTGTTCAACGATTAAAAC | |
| *Oreobates quixensis* | TWQ_037 | 2020 | AAAACATCGCCTCTTGTTCTAAATAAGAGGTCCAGCCTGCCCAGTGACTTGTTTAACGGCCGCGGTATCCTAACCGTGCGAAGGTAGCGTAATCACTTGTTTTTTAAATAAAGACCTGTATGAAAGGCACCACGAGAGCCAAACTGTCTCCTTTTTCCTATCAGTGAAACTAATCTTCCCGTGAAGAAGCGGGAATTTTTTAATAAGACGAAAAGACCCTATGGAGCTTTAAACTTCACAACACCTGTTTCTCAATCTTTAATATTCTCTCTAAACACCCTGTTTATGAACTTTCGGTTGGGGCGACCACGGAGAATAACTCACCCTCCGTAATGAGTAACAAAAAGCAACAACTTTATTCATCAGTAAAACTGACATCCATTGACCCAAATCTTTGATCAACGAACCAAGTTACCCTAGGGATAACAGCGCTATCCACTTTAAGAGCCCCTATCGACAAGTGGGCTTACGACCTCGATGTTGGATCAGGGTATCCTGGTGGTGCAGCCGCTACTAACGGTTCGTTTGTTCAACGA | |
| *Osteocephalus buckleyi* | KF002027 | 2013 | TATAAGAGGTCCAGCCTGCCCAGTGACTTTGTTCAACGGCCGCGGTATCCTAACCGTGCGAAGGTAGCGTAATCACTTGTCTTTTAAATGAGGACTTGTATGAATGGCATCACGAGGGTTATACTGTCTCCTTTCTCTAATCAGTGAAACTAATCTTCCCGTGAAGAAGCGGGAATAAGAATATAAGACGAGAAGACCCTATGGAGCTTTAAACTCAATAACAAATACTAACCTTACTTTAACTTCCAGAACATTGACACTAATCTTAGTACTATGATTATTAGTTTTAGGTTGGGGTGACCGCGGAGTAAAAATTAACCTCCACATTGAACAGGACCTTTCCTTGAGCTAAGAGCTACAACTCTAAACACCAACAAATTGACATTAATTGACCCAATATTTTGATCAACGAACCAAGTTACCCTAGGGATAACAGCGCAATCCACTTCAAGAGCTCCTATCGACAAGTGGGTTTACGACCTCGATGTTGGATCAGGGTATCCCAGTGGTGCAGCCGCTACTAAAGGTTCGTTTGTTCAACGATTAAAACCCT | |
| *Phyllomedusa tarsius* | KY576698 | 2017 | CGCCTCCTGCTAACCTATAGGAGGTCCAGCCTGCCCAGTGACTCTGTTCAACGGCCGCGGTATCCTAACCGTGCGAAGGTAGCGTAATCACTTGTTCTTTAAATGAGGACTAGTATGAATGGCACCACGAAGGTTATACTGTCTCCTTTTCCCAATCAGTGAAACTAATCTCCCCGTGAAGAAGCGGGGATTAACTTATAAGACGAGAAGACCCTATGGAGCTTCAAACTTTTCAGCAATTATCTTCACTTATATAAGTTTCCCTAAACAATGAAATTAATCTAGATAATATGTATGCAAGTTTTCGGTTGGGGTGACCGCGGAGCAAAACTTACCCTCCGCAATGAATTAGAATTTTACCTCTAAACAAAGAATTACAATTCTATGTATCAAAAAACTGACACAATTGACCCAATACTTTGATCAACGAACCAAGTTACCCTAGGGATAACAGCGCAATCCATCTCAAGAGCTCTTATCGACAGATGGGTTTACGACCTCGATGTTGGATCAGGGTGTCCCAGTGGTGCAGCCGCTACTAAAGGTTCGTTTGTTCAACGATTGAAACCCT | |
| *Phyllomedusa tomoptera* | EF376077 | 2007 | AAGAGGTCCAGCCTGCCCAGTGACTCTGTTTAACGGCCGCGGTATTCTAACCGTGCGAAGGTAGCGTAATCACTTGTTCTTTAAATGAGGACTAGTATGAATGGCATCACGAGGGTTATACTGTCTCCTTTTCCAAATCAGTGAAACTAATCTCCCCGTGAAGAAGCGGGGATAAAAATATAAGACGAGAAGACCCTATGGAGCTTAAAACTTTCAACAATTATATTTTATTTATAAATCTAAAGGTAATCAAACTAATCCTTATAATATGTTTGTTAGTTTTCGGTTGGGGTGACCGCGGAGTAAAACCTAACCTCCATATTGAATTAAATTATTTATTAGCAAAAAACTACAATTTTATGCATCAAAAAATTGACATTATTGACCCA | |
| *Pristimantis altamazonicus* | MF118696 | 2003 | ATAAGAGGTCCAGCCTGCCCAGTGATCTATTCAACGGCCGCGGTATTCTAACCGTGCGAAGGTAGCGTAATCACTTGTCTTCTAAATAAAGACCAGTATGAAAGGCATCACGAAGACTAAGCTGTCTCCTCTCTCCAATCAGTGAACCTAATCTCCCCGTGAAGAAGCGGGGATCCAATTATAAGACGAGAAGACCCTATGGAGCTTTAAACATCGTAACATCTGCCACCGCTTCTACCCACTCCGGTGTACCCCCTAAACGGCACCCTGATTACATGTTTTTGGTTGGGGTGATCGCGGAGAAAAACTCATCCTCCACAATGAAAGGGACCTCCCCTAAACAATAAGTTACAACTTTATGTATCAAGAAATTGACATCCATTGACCCAATAACTGATTAACGAACCAAGTTACCCTAGGGATAACAGCACAATCCACTTTGAGAGCCCGTATCGACAAGTGGGCTTATGACCTCGATGTTGGATCGGGATATCCCAGTGGTGCAACCGCTACTAAAGGTTCGTTTGTTCAACGATTAAAACCCC | |
| *Pristimantis altamnis* | KP064155 | 2015 | TGCAAAGGTAGCGTAATCACTTGTCCTCTAAATAGGGACCAGTATGAAAGGCATCACGAGGACTAAACTTCTCCTTTTTCTAATCAGTGAACCTAATCTCCCCGTGAAAAAGCGAGGATCCTCATATAAGACGAGAAGACCCTATGGAGCTTAAAACATAACAATATTTGGCATTATTATTGTGTGTTTTGGTTGGGGCGACCGCGGAGAAAAACCTATCCTCCTCAATGAACAAACAACAAGTTACAACTTTAAGTATCAAAAAATTGACATCCTTGACCCAATAAATTGATTAATGAACCAAGTTACCCTAGGGATAACAGCACAATCCACTTTGAGAGCTCCTATCGACAAGTGGGCTTATGACCTCGATGTTGGATTAGGGTATCCTAGTGGTGCAGCCGCTACTAAAGGTTCGTTTGTTCAACGATTAAAACCCCACGTGATCT | |
| *Pristimantis brevicrus* | MF118702 | 2017 | ATAAGAGGTCCAGCCTGCCCAGTGACTCATTCAACGGCCGCGGTATACTAACCGTGCGAAGGTAGCGTAATCACTTGTCTTCTAAATAAAGACCAGTATGAAAGGCATCACGAAGGCTAAACTGTCTCCTCTCTCCAATCAGTGAACCTAATCTCCCCGTGAAGAAGCGGGGATCCCATTATAAGACGAGAAGACCCCATGGAGCTTAAAACACCATAACACCTGCCCCAATTCTCAACCACTCCGATGTTTCCATGTAACGGCACCCTGATTATGTGTTTTTGGTTGGGGTGACCGCGGAGAAAAACTCATCCTCCACAATGAAAGGGGCCCTCCCCTAAACAATAAGTTACAACTTTATATATCAAAAAATTGACATTCATTGACCCAACAAATTGATTAACGAACCAAGTTACCCTGGGGATAACAGCACAATCCACTTTGAGAGCCCCTATCGACAAGTGGGCTTATGACCTCGATGTTGGATCGGGATATCCTAGTGGTGTAACCGCTACTAAAGGTTCGTTTGTTCAACGATTAAAATCCCACGTGATC | |
| *Pristimantis conspicillatus* | MH516169 | 2018 | GAGGTCCAGCCTGCCCAGTGACGTTCAACGGCCGCGGTATCCTAACCGTGCAAAGGTAGCGTAATCACTTGTCTTTTAAATAAAGACTAGTATGAAAGGCACCACGAGAATTAAACTGTCTCCTTTTTCCAATCAATGAACCTAATACCCCCGTGAAGAAGCGGGGATTTTACAATAAGACGAGAAGACCCTATGGAGCTTTAAACAATTATATGTTTTTGGTTGGGGTGACCGCGGAGAAAAACCTATCCCCCGCGATGAAAAACAAGCCACAACTTTATGTATCAACAAATTGACACCATTGACCCAATATATTGATTAACGAACCAAGTTACCCTAGGGATAACAGCGCAATCCACTTCAAGAGCTCCTATCGACAAGTGGGCTTACGACCTCGATGTTGGATCAGGGTATCCTAGTGGTGCAGCCGCTACTAAGGGC | |
| *Pristimantis diadematus* | MW567354 | 2022 | AGCCTTTCTCCCCACTCCCCCCTTTAATAAGCCTTAACTAAAATATTTTAACAGCCTAGTAAAGAAGATTAAAAAACTTTTAAGAGCTATAAATTGGTACCGCAAGGGATCGATGAAATAAAAATGAAATAAACCTCAAGCACAAAAAAGTAAAGATTCATCCTTGTACCTTTTGCATCATGGTTTAGCTAGTCTAATTAGGCAAAAAGACCTTAAGTCTAATTTCCCGAAACTAAGTGAGCTATTTTAAGGCCGCTTACTGAGCTAACCCGTCCATGTTGCAAAATGGTGGGAAGACCTTAAAATAGAGGTGACAAGTCTAACGAACTTAGTTATAGCTGGTTTCTCAAGAAAAGAATTTTAGTCCAACCTAAAGTAAATTCAAACTTCACCCGAATTAGCCTCCTTACTTTAGAGTAATTCAAACGAGGTACAGCCCATTTGAAACAGGATACAACCTTCACTAACGGGTAACATTCAGTATATACCTTAAAGTAGGCCTAAAAGCAGCCATCTTTTAAAAAGCGTCAAAGCTTAACCGTACATGAAAAAAATCTAAAAATTCACATTGACCCCTTTAGTTCTACTGAGAATTTCTATAACCCTATAGAACCTTTTATGCTAAAACTAGTAACAAGAGGAAACCCCTCTCCCCAATGCAAACTTAAGCCAGTAAGAACCCCCCACTGGCAATTAACAAAAAAGTAGCAACCCTCACTAGAAAACCCTACTTACCAAACTGTTAACCTAACACCAGAGCATTTCAAGAAAGATTAAAAGAAAAAGAAGGAACTCGGCAAACATTAGCCTCGCCTGTTTACCAAAAACACCGCCTCTTGTTATCATAAGAGGTCCAGCCTGCCCAGTGATTTTATTCAACGGCCGCGGTATTCTAACCGTGCAAAGGTAGCGTAATCACTTGTTTTCTAAATAAAGACTAGTATGAAAGGCATCACGAAGACTAAGCTGTCTCCTTTCTCCAATCAGTGAACCTAATCTCCCCGTGAAG | |
| *Pristimantis eriphus* | DQ195458 |  | TCCAGCCTGCCCAGTGACTCTTGTTCAACGGCCGCGGTATCCTAACCGTGCAAAGGTAGCGTAATCACTTGTTTTCTAAATAAAGACCAGTATGAAAGGCATCACGAGGACTAAACTGTCTCCTTTTTTCAATCAGTGAATCTAATCTCCTCGTGAAGAAGCGAGGATTCTAATATAAGACGAGAAGACCCTATGGAGCTTAAAACATCATTACACTTTCCACCGCATTTCCATGCTCCGGCACCTTCAGCACTGGCACACTGATTATATGTTTTTGGTTGGGGCGACCGCGGAGAAAAACCCATCCTCCGCGATGAAAAGGGCTTCCCCCTTAAACAACAAACCACAACTTTATGTATCAACAAATTGACATCTATTGACCCAATATATTGATTAACGAACCAAGTTACCCTAGGGATAACAGCGCAATCCACTTTAAGAGCCCCTATCGACAAGTGGGCTTACGACCTCGATGTTGGATTAGGGTACCCCAGTGGTGCAGCCGCTACTAAAGGTTCGTTTGTTCAACGATTAAAACCCC | |
| *Pristimantis kichwarum* | EU130603 | 2007 | CAGTGACTTTATTCAACGGCCGCGGTATCCTAACCGTGCAAAGGTAGCGTAATCACTTGTCCCCTAAATAGAGACCAGTATGAAAGGCATCACGAGGGCTAAACTGTCTCCTTTTTCTAATCAGTGAACCTAATCTCCCCGTGAAAAAGCGGGGATCCCCCTATAAGACGAGAAGACCCTATGGAGCTTAAAACATAACAATACCTGCCTCACCTAATATACCCAGATTTTCCCCCTCCCCCACGTTCTTATTGTGTGTTTTTGGTTGGGGCGACCGCGGAGAAAAACCTATCCTCCTCAATGAAATGGGACTCCCCTAAACAACAAGCTACAACTTTAAGTATCAAAAAATTGACATCCCTTGACCCAATAAATTGATTAATGAACCAAGTTACCCTAGGGATAACAGCACAATCCACTTTGAGAGCTCCTATCGACAAGTGGGCTTATGACCTCGATGTTGGATTAGGGTATCCTAGTGGTGCAGCCGCTACTAAAGGTTCGTTTGTTCAACGATTAAAACCCCACGTGATCT | |
| *Pristimantis lanthanites* | HMOA1942 | 2018 | CGGCCGCGGAACCCTAACCGTGCGAAGGTAGCGTAATCACTTGTCCTATAAATGAGGACTAGTATGAAAGGCTTCACGAGGGCTAAACTGTCTCCTTCTTCTGATCAGTAAACCTAATCTCCCCGTGAAAAAGCGGGATTAACCACATAAGACGAGAAGACCCCATGGAGCTTAAAACACTTCTACACTTGTCACAAACCTATAATAACTACTATGACACCCTGTTTAAATGTTTTCGGTTGGGGTGACTGCGGGGAATAACTAAACCCCCGCGATGAAAGGGTACATCCCTAAACTATAAGTTACAACTTTAAATATCAGAAAACTGACATCTTTGACCCAATTATTTGATCAACGAACCAAGTTACCCTGGGGATAACAGCGCAATCCACCTCAAGAGCCCTTATCGACAGGTGGGCTTACGACCTCGATGTTGGATCAGGGTATCCTAGTGGTGCAGCCGCTACTAAAGGTTCGTTTGTTCAACGATTAAAACCCTACGTGATCTGAGTTCAGACCG | |
| *Pristimantis librarius* | JN991451 | 2011 | CACCTTTTGATAAGAGGCCCAGCCTGCCCAGTGACGTTCAACGGCCGCGGTATTCTAACCGTGCAAAGGTAGCGTAATCACTTGTCTTCTAAATAAAGACTAGTATGAAAGGCACCACGAGGACTAAACTGTCTCCTTTTTCTAATCAGTGAATCTAATCTCCCCGTGAAGAAGCGGGGATAAAAATATAAGACGAGAAGACCCTGTGGAGCTTAAAACATTTAAACACTTGCATCCTGATTTCATGTTTTTGGTTGGGGTGACCGCGGAGAAAAACCTATCCTCCACAGTGAACAGCAATAAGTTACAACCTTGCGCATTAAAAAATTAACACTATTGACCCAATATTTTGATCAATGAACCAAGTTACCCCAGGGATAACAGCACAATCCACTTCGAGAGCCCCTATTGACAAGTGGGCTTATGACCTCGATGTTGGATCGGGGTATCCTAGTGGTGCAGCCGCTACTAAAGGTTCGTTTGTTCAACGATTAAAACCTC | |
| *Pristimantis malkini* | TWQ_016 | 2020 | AACATCGCCTCTTGACTCTTATAAGAGGTCTAGCCTGCCCAGTGACCTAGTTCAACGGCCGCGGTATCCTAACCGTGCGAAGGTAGCGTAATCACTTGTCTTCTAAATAAAGACTAGTATGAAAGGCACCACGAGAACTAAACTGTCTCCCTTTTCCAATCAGTGAACCTAATGCCCCCGTGAAGAAACGGGGATTTTACAATAAGACGAGAAGACCCTATGGAGCTTTAAACATCATAATCACCTGCCTACTAAACCAAGATACTCAGGTCCCCCACCCAAACCCATGATGATTATATGTTTTTGGTTGGGGTGACCGCGGAGAAAAACACATCCCCCGCGATGAAAGCCCACCCAAACAACAAGCCACAGCTTTATGTATCAACAAATTGACACTATTGACCCAATATATTGATTAACGAACCAAGTTACCCTAGGGATAACAGCGCAATCCACTTCAAGAGCCCCTATCGACAAGTGGGCTTACGACCTCGATGTTGGATCAGGGTATCCTAGTGGTGCAGCCGCTACTAAGGGCTCGTTTGTTCAACGATTAAAACCCTACGTGA | |
| *Pristimantis variabilis* | TWQ_018 | 2020 | TCGCCTCCTGTTCTCATAGGAGGTCCAGCCTGCCCAGTGACTTAATTTAACGGCCGCGGTATCCTAACCGTGCGAAGGTAGCGTAATCACTTGTCTTCTAAATAAAGACTTGTATGAAAGGCATCACGAGGGCTTAACTGTCTCCTTTCTCCAATCAGTGAACCTAATCTCCCCGTGAAGAAGCGGGGATAACTATATTAGACGAGAAGACCCTATGGAGCTTTAAATACTATAACATATGCTTTCCCCCCTATATCACCGAGGTGCCTCACCTTTCACAGCTAATTGATTATATATTTTTGGTTGGGGTGACCGCGGAGAATAAACCATCCTCCACAATGAATGGGGATCCCCCCCAATAAACAAGCCACAACTTTACCCATCAATAAATTGACTCTCATGACCCAATATTTGATCAACGAACCAAGTTACCCTAGGGATAACAGCGCAATCCACTTTAAGAGCTCCTATCGACAAGTGGGCTTACGACCTCGATGTTGGATCAGGGTATCCTAGT | |
| *Rana palmipes* | DQ347321 | 2006 | CGCCTCTTGACAAAATATAAGAGGTCCAGCCTGCCCAGTGATAAATTTAACGGCCGCGGTATCCTAACCGTGCGAAGGTAGCATAATCATTTGTTCTTTAAATGGGGACTTGTATCAACGGCACCACGAGGGCCATACTGTCTCCTTTCTCTAGTCAGTGAAACTGATCTCCCCGTGAAGAAGCGGGGATTTAACTATTAGACGAGAAGACCCCATGGAGCTTAAAACTCACTATATAATTCTGTACCTCATATCACCTTAATTCAGAATCCTATATGCTAGTTTTAGGTTGGGGGGACCTCGGAGTATAATTTAACCTCCATAACAAATGGGCTAATACCCTTATCCAAGATAAACACCTCTAAGAATTATTAAATTAATGTTTAATGACCCGATATATTCGATCAATGAACCAAGTTACCCTGGGGATAACAGCGCAATCTACTTCAAGAGTCCATATCGACAAGTAGGTTTACGACCTCGATGTTGGATCAGGGTATCCTAGTGGTGCAACCGCTACTAATGGTTC | |
| *Ranitomeya variabilis* | JN651262 | 2011 | TAGCGTAATCACTTGTTCTTTAATTGAGGACTAGTATGAACGGCCCCACGAGGACTACACTGTCTCCTTTTTCTAATCAATGAAACTGATCTCCTCGTGAAGAAGCGAGGATGAATATATAAGACGAGAAGACCCTATGGAGCTTTAAACAACCGGAGTACTTGCTATTCATTTTATATCTTCTGAGAACTAAACAAATTAAAGCATCTTACTCCCAAGTTTTAGGTTGGGGTGACCACGGAGAAAAAATTAACCTCCACGCTGTAAGAAGTTCACCATCTTCTAAGCTTAGAGTCACATCTCCAAGCATCAACACATTGACTCCCATTGACCCAATAATTTGATCAACGAACCAAGTTACCCTAGGGATAACAGCGCAATCTACTTTAAGAGCTCATATCGACAAGTAGGTTTACGACCTCGATGTT | |
| *Ranitomeya ventrimaculata* | JN635822 | 2011 | TAGCGTAATCACTTGTTCTTTAATTGGGGACTAGTATGAACGGCCCCACGAGGACTACACTGTCTCCTTTTTCTAATCAATGAAACTGATCTCCTCGTGAAGAAGCGAGGATAATTATATAAGACGAGAAGACCCTATGGAGCTTCAAACAACCGGGATACTTGCTGATTCTTAATCTATTTCCTGAAAACTAAACAAATTAAAGCATCTTACCCCCTAGTTTTAGGTTGGGGTGACCACGGAGAAAAAATTATCCTCCACGCTGTAAGAAGCCTCCCATCTTCTAAGCTAAGAGATTACACTCTCTAAGCATCAACACATTGACTCCCATTGACCCAATAATTTGATCAACGAACCAAGTTACCCTAGGGATAACAGCGCAATCACTTTAGAGCTCATATCGACAAGTAGGTTTACGACCTCGATGTT | |
| *Rhaebo ecuadorensis* | TWQ_3639 | 2020 | ACATCGCCTCTTGATTAGTTATAAGAGGTCCAGCCTGCCCAGTGACCCTGTTCAACGGCCGCGGTATCCTAACCGTGCGAAGGTAGCGTAATCACTTGTTCTTTAAATAAGGACTAGTATGAATGGCACCACGAGGGCTACACTGTCTCCTTTTTCTAATCAGTGAACCTAATCTTCCCGTGAAGAAGCGGGAATAAACCTATAAGACGAGAAGACCCTATGGAGCTTTAAACAATATAGCATTTGCCATATAACATAAAAATTTCCGAATATTCACTTCTTAAAGCAGTATGACTATAAGTTTTTGGTTGGGGTGACCGCGGAGCATAACACAACCTCCATGTTGAATGAAGATTATTCTAAGCCGCGAACCACACTTCTAAGCATCAAAATACTGACACCAATTGACCCAATATATTTGAACAACGAACCAAGTTACCCTAGGGATAACAGCGCAATCCACTTCAAGAGCTCCTATCGACAAGTGGGTTTACGACCTCGATGTTGGATCAGGGTCTCCCAGTGGTGTAGCCGCTACTAAAGGTTCGTTTGTTCAACGATTAAAACCCTACGTGATC | |
| *Rhinella dapsilis* | KR012641 | 2015 | GCTCAATTTCACAAACATTATCTTAATCCTCCATCTTTATTGAGTAATTCTATAATTTTATAGAAAATTTTATGTTAAAACTAGTAACAAGAAGAAGACCTTCTCTTTAATGTAAGTGTCAATCAGAAAGGACAAACCACTGATATTTAACATCACTGAACCAAAAACAGTAACTCATCAAGAAAATCCTGTTTTTATTTATGTTAACCTAACACAAGAGCATTCTAAGAAAGATTAAAAGAAAAAGAAGGAACTCGGCAAATATTAACCTCGCCTGTTTACCAAAAACATCGCCTCTTGAACTTTATAAGAGGTCCAGCCTGCCCAGTGACTCTGTTCAACGGCCGCGGTATCCTAACCGTGCGAAGGTAGCGTAATCACTTGTTCTTTAAATGCGGACTAGTATGAATGGCACTACGAGGGTTATACTGTCTCCTTTTTCTAATCAGTGAAACTAATTTTCCCGTGAAGAAGCGGGAATATAACTATAAGACGAGAAGACCCTATGGAGCTTTAAACAACCCAGCAATTACTTCTAAAATTGTAAATTTCAGAATATTCATTTCTNTAAGTAATGTGTCTGCAAGTTTTTGGTTGGGGTGACCATGGAGAATAACATAACCTCCATATTGAATGAATTCCCTTCTAAGCTGAGAATTACCAATTGAAGCATCAACACACTGACATCTATTGACCCAATATATTTGATCAACGAACCAAGTTACCCTAGGGATAACAGCGCAATCCACTTCAAGAGCCCCTATCGACAAGTGGGTTTACGACCTCGATGTTGGATCAGGGTATCCCAGTGGCGCAGCCGCTACTAAAG | |
| *Rhinella festae* | KR012624 | 2015 | TTATAGAATATTTTATGTTAAAACTAGTAACAAGAAAANGACTTTCTCTTTAATGCAACTATAAATCAGAAAGGACAAACCACTGATATTTAACATCATTGAACCGAAAGTAAAAACTCATCAAGAAAACCCTACTCTCATTTATGTTAACCTAACACAAGAGCATTACAAGAAAGATTAAAAGAAAAAGAAGGAACTCGGCAAATATTAACCTCGCCTGTTTACCAAAAACATCGCCTCTTGAATCCTATAAGAGGTCCAGCCTGCCCAGTGACTTTGTTCAACGGCCGCGGTATCCTAACCGTGCGAAGGTAGCGTAATCACTTGTTCTTTAAATAAGGACTAGTATGAATGGCTCCACGAAGGTTATACTGTCTCCTTTTTCTAATCAGTGAAACTAATCTTCCCGTGAAGAAGCGGGAATACTAATATAAGACGAGAAGACCCTATGGAGCTTAAAATAACTCAACATTTGCTTANAACATTAAAAATTTCAGAACTTTGACTTCTACTAGCAATATGATTGTAAATTTTTGGTTGGGGTGACCACGGAGAACAATACAACCTCCATGCAGAAAGAATTTTCTTCTAAGCTAAGAATTACTTGTCTAAGCATCAACACATTGACATTCCTTGACCCAATATAATTGATCAACGAACCAAGTTACCCTAGGGATAACAGCGCAATCTACTTCAAGAGCTCCTATCGACAAGTAGGTTTACGACCTCGATGTTGGATCAGGGTATCCCAGTGGTGTAGCCGCTACTAAA | |
| *Rhinella margaritifera* | HMOA1973 | 2018 | AAAAACATCGCCTCTTGAACTTTATAAGAGGTCCAGCCTGCCCAGTGACTCTGTTCAACGGCCGCGGTATCCTAACCGTGCGAAGGTAGCGTAATCACTTGTTCTTTAAATGCGGACTAGTATGAATGGCACTACGAGGGTTATACTGTCTCCTTTTTCTAATCAGTGAAACTAATTTTCCCGTGAAGAAGCGGGAATATAACTATAAGACGAGAAGACCCTATGGAGCTTTAAACAACCCAGCAATTACTTCTAAAATTGTAAATTTCAGAATATTCATTTCTTAAGTAATGTGTCTGCAAGTTTTTGGTTGGGGTGACCATGGAGAATAACATAACCTCCATATTGAATGAATTCCCTTCTAAGCTGAGAATTACCAATTGAAGCATCAACACACTGACATCTATTGACCCAATATATTTGATCAACGAACCAAGTTACCCTAGGGATAACAGCGCAATCCACTTCAAGAGCCCCTATCGACAAGTGGGTTTACGACCTCGATGTTGGATCAGGGTATCCCAGTGGCGCAGCCGCTACTAAAGGTTCGTTTGTTCAACGATTAAAACCCTACGTGATCTG | |
| *Rhinella marina* | TWQ_2728 | 2020 | CATCGCCTCTTGACTCTTATAAGAGGTCCAGCCTGCCCAGTGACCATGTTCAACGGCCGCGGTATCCTAACCGTGCGAAGGTAGCGTAATCACTTGTTCTTTAAATRCGGACTAGTATGAATGGCACCACGAAGGTTATACTGTCTCCTTTTTCTAATCAGTGAAACTAATCTCCCCGTGAAGAAGCGAGGATAYACCTATAAGACGAGAAGACCCTATGGAGCTTTAAACAACACAGCAATTACCTGCTAAACTAAGAAGTTTCTGAACATTTTCAATCTTTTAAGTAATCTGACTGCAAGTTTTTGGTTGGGGTGACCACGGAGCATAACACAACCTCCAYGTTGAAAGAATTCTTTCTAAGCCAAGAACAACCTTTCTAAGCATCAATATATTGACATCCATTGACCCAATACATTTGACCAACGAACCAAGTTACCCTAGGGATAACAGCGCAATCCACTTCAAGAGCTCCTATCGACAAGTGGGTTTACGACCTCGATGTTGGATCAGGGTA | |
| *Rulyrana flavopunctata* | HMOA1986 | 2018 | AAAAACATCGCCTCTTGAACAAATATAAGAGGTCCAGCCTGCCCAGTGACTCTGTTCAACGGCCGCGGTATCCTAACCGTGCGAAGGTAGCGTAATCACTTGTTCTTTAAATAAGGACTAGTATGAATGGCACCACGAAGGTTATACTGTCTCCTCTTTTTAATCAGTGAAACTAATCTCCCCGTGAAGAAGCGGGGATAAAACTATAAGACGAGAAGACCCTATGGAGCTTTAAACCATGTAGCACTTGCACCTATCAAACACAAATTTCAGAAAAATACACTTCACCCATGCAAGATTGCTGCCAGTTTTAGGTTGGGGTGACCGCGGAGCAAAAATAAACCTCCACATTGAAAGGAACCCACTCCTTAGCCAAGAGTTACACCTCTACGCACCATAAAAATGACATTAATTGACCCAATAATTTGATCAACGAACCAAGTTACCCTAGGGATAACAGCGCAATCCACTTTAAGAGCTCCTATCGACAAGTGGGTTTACGACCTCGATGTTGGATCAGGGTATCCCGGTGGTGCAGCCGCTACCAAAGGTTCGTTTGTTCAACGATTAAAACCCTACGTGATCT | |
| *Scinax aff cruentomma* | TWQ_012 | 2020 | CGTAATCACTTGTTCTTTAAATGGGGACTAGTATGAATGGCACCACGAGGGTTATACTGTCTCCTTTCTCTGATCAGTGAAACTAATCTCCCCGTGAAGAAGCGGGGATACAGCTATAAGACGAGAAGACCCTATGGAGCTTTAAATCTCTTTTCAAACGCTAAAATACTTATACAAGTCCCAGACTTACCCTCACACTTACCTAGCGCCATGAAAATGATTTTAGGTTGGGGTGACCGCGGAGCACAAAAAAACCTCCACGCTGAACTAGACGTCCAAACCTTGAGCTAAGGGCTACGACCCGATGCACTAAAAAATTAACACCAATTGACCCAATATTTATTTGATCAACGAACCAAGTTACCCTAGGGATAACAGCGCAATCCATTTCAAGAGTCCATATCGACAAATGGGTTTACGACCTCGATGTTGGATCAGGGTCTCCCAGTGGTGCAGCCGCTACTAATGGTTCGTTTGTTCAACGATTAAAACCCTA | |
| *Scinax garbei* | TWQ_008 | 2020 | AAAAACATCGCCTCTTGCCTAAAATAAGAGGTCCAGCCTGCCCAGTGACACATGTTCAACGGCCGCGGTATCCTAACCGTGCGAAGGTAGCGTAATCACTTGTTCTTTAAATGAGGACTAGTATGAATGGCATCACGAGGGTTATACTGTCTCCTTTTTCTAATCAGTGAAACTAATCTCCCCGTGAAGAAGCGGGGTTGAAATTATAAGACGAGAAGACCCTATGGAGCTTTAAATTTCCACTCAAATGCTTATTAACCTTTATACTTCTTAGTATTATTAAAAAGTACCTTAGCATTATGAGCCAAAATTTTAGGTTGGGGTGACCGCGGAGTACAAAATAACCTCCACATTGAAACAGGCTTACAAACCCTGAGCAACGGACAACAATCCTATGCACTAAAAAACTAACATCCATTGACCCAAGTTTTTGATCAACGAACCAAGTTACCCTAGGGATAACAGCGCAATCCTCTTCAAGAGTTCCTATCGCCAAGAGGGTTTACGACCTCGATGTTGGATCAGGGTATCCCAGTGGTGCAGCCGCTACTAATGGTTCGTTTGTTCAACGATTAAAACCCTACGTGATCT | |
| *Scinax ruber* | TWQ_2022 | 2020 | AAAACATCGCCTCTTGAACAAAAATAAGAGGTCCAGCCTGCCCAGTGACTAATGTTCAACGGCCGCGGTATCCTAACCGTGCGAAGGTAGCGTAATCACTTGTTCTTTAAATGAGGACTAGTATGAATGGCACCACGAGGGTTATACTGTCTCCTCTTTCTAATCAGTGAAACTAATCCCCCCGTGAAGAAGCGGGGATTAAACTATAAGACGAGAAGACCCTATGGAGCTTTAAATTTACCCTCAAACGCCAAAATCATACAGCAAGTTTTAAACTTCACCACACGAACTTTCGCGCCATGAGGGCAACTTTTAGGTTGGGGTGACCATGGAGTATAAAATAACCTCCATACTGAACAGGCCTCCAAGCCTCGAACCAAGAGCCACGACCCGCCGCACTAAAATATTAACATCCATTGATCCAATGTATTTTGATCAACGAACCAAGTTACCCTAGGGATAACAGCGCAATCTACTTCAAGAGTTCCTATCGACAAGTGGGTTTACGACCTCGATGTTGGATCAGGGTGTCCCAGTGGTGCAGCCGCTACTAATGGTTCGTTTGTTCAACGATTAAAACCCTAC | |
| *Siphonops annulatus* | EU753986 | 2008 | TGCCTGCACAGTGATATTCTTTTAACGGCCGCGGTATTCTGACCGTGCAAAGGTAGCATAATCACTTGTTTTTTAAATAAAGACTAGTATGAATGGCAAGACGAAAGTTTAACTGTCTCCTTTGTCTAGTCAATGAAATTGATCTCCTGGTACAAAAGCCAGGATAACATAATTAGACGAGAAGACCCTATGGAACTTAAAACACGCATCCACCCATACGTTTATAAAACATCTCATATATTGGACCGCTGTTTTTGGTTGGGGCAACCACGGAGAAAAACAATCCTCCTAGAGATTAAAATTAAGTAATACAATACAAAATTCCAATACCATGGACATGATCCATATTCATGATCAACGAACCAAGCTACCCTAGGGATAACAGCGCTATCTTCTCCAAGAGTCCATATCCACGAGAAGGTTTACGACCTCGATGTTGGATCAGGATATCCAGACGGCGCAGCCGCCGTCAAAGGTTTGTTTGTTCAACAATTAAAATCCTACGTGATCTGAGTTCAAAACCG | |
| *Teratohyla midas* | MT225195 | 2020 | CCCCTAAAGAACCCTCAAATAATACTGAGTAATTCTATATTTATATAGAAGATTTTATGTTAAAACTAGTAACAAGAAGAAGAACTTCTTACATAATGCAAGTGTAAACCAGACAGGACACCCCACTGACAATTACCACCAATGAAGAAAAAGTAGCAACTCCACAAGAAAACCCTACTAAACCAAGTGTTAACCTAACACCAGAGCATTACAAAAAAGATTAAAAGAAAGAGAAGGAACTCGGCAAATATTAACCCCGCCTGTTTACCAAAAACATCGCCTCTTGATAAAATATAAGAGGTCCAGCCTGCCCAGTGACTCTGTTCAACGGCCGCGGTATCCTAACCGTGCGAAGGTAGCGTAATCACTTGTTCTTTAAATGAGGACTAGTATGAATGGCACCACGAAGGTTATACTGTCTCCTCTTTCCAATCAGTGAAACTAATCTCCCCGTGAAGAAGCGAGGATAAACCTATAAGACGAGAAGACCCTATGGAGCTTTAAACCAAGTAGCACTTGCATCAGTTTAATATAAATTTCAGAAAAACCTCACCCACCCATGCAAGATTATTACTAGTTTTAGGTTGGGGTGACCGCGGAGCAAAAGACAACCTCCACATTGAAAGGAACCCACTCCTCAGCCAAGAGTTACACCTCTATGCACCATTATAATGACATTAATTGACCCAATATTTTGATCAACGAACCAAGTTACCCTAGGGATAACAGCGCAATCCACTTCAAGAGCTCCTATCGACAAGTGGGTTTACGACCTCGATGTTGGATCAGGGTATCCCAGTGGTGTAGCCGCTACTAATGGTTCGTT | |

**Table S3**. **List of 20 species collected in the Tena River micro-basin, Napo, Ecuador and their respective sequences.**

|  | Species | Code | Sampling year | Sequences |
| --- | --- | --- | --- | --- |
| *1* | *Adenomera andreae* | TWQ_019 | 2020 | CGCCTCTTGCCTCAAATAAGAGGTCCAGCCTGCCCAGTGACACTGTTCAACGGCCGCGGTATCCTAACCGTGCGAAGGTAGCGTAATCACTTGTTCTTTAAATGAGGACTAGTATGAAAGGCATCACGAGAGTCATACTGTCTCCTTTTTCCAATCAGTTAAACTAATCTCCCCGTGAAGAAGCGAGGATAATTCTATAAGACGAGAAGACCCTATGGAGCTTTAAACAAATAACACTTACCTACCCCCTCCTTTAATTTCAGAAAAATCTCCCACTTTGGCATGATACTTATAAGTTTTTGATTGGGGTGATCACGGAACAAAAAACAACCTCCGCAATGAAAGACACTCCCCTAACTAAGTTAAGAACTACAGCTCTATACATCAACAAATTGACACTAATTGACCCAATATATTGATCAATGAACCAAGTTACCCTAGGGATAACAGCGCAATCTACTTCAAGAGCTCCTATCGACAAGTGGGTTTACGACCTCGATGTTGGATCAGGGTATCCTAATGGTGTAGCCGCTATTAAAGGTTCGTTTGTTCAACGATTAAAACCCTACGT |
| *2* | *Allobates aff insperatus* | TWQ_033 | 2020 | AACATCGCCTCTTGACTAACCATAAGAGGTCAAGCCTGCCCAGTGACATTGTTCAACGGCCGCGGTATCCTAACCGTGCGAAGGTAGCGTAATCACTTGTCCTTTAAATAGGGACTAGTATGAATGGCTTCACGAAAGCTATACTGTCTCCTTTATTTAATCAGTAAAACTAATTCCCCCGTGAAGAAGCGGGGATACACCTATAAGACGAGAAGACCCTGTGGAGCTTTAAACACTTTTTAAACATTTGAATTTACACTCTTATTCTATCTCTACTAAATCACTCATTTTGTTTTAAGTTTTAGGTTGGGGTGACCACGGAGAATAAATTAACCTCCACGTAGAAAGAACCTTATTTTCTAAGCAAAAAATCACATTTTTATGCATCAAAATATTTGACCTAAATTGACCCAATTTTTTGATCAACGAACCAAGTTACCCTAGGGATAACAGCGCAATCCGCTTTAAGAGCTCCTATCGACAAGCGGGCTTACGACCTCGATGTTGGATCAGGGTA |
| *3* | *Boana geographica* | TWQ_009 | 2020 | AACATCGCCTCTTGAGTAAAATAAGAGGTCCAGCCTGCCCAGTGAAATTATTTAACGGCCGCGGTATCCTAACCGTGCGAAGGTAGCGTAATCACTTGTTCTTTAAATGAGGACTAGTATGAATGGCACCACGAGGGTTCTACTGTCTCCTTTTTTAAATCAGTGAAACTAATCTCCCCGTGAAGAAGCGGGGATTTTTTTATAAGACGAGAAGACCCTATGGAGCTTTAAACAAACATCATTTGCTGCACATAACACTTTTACCTTTAATAAATATAGCATTATGACCTTTAGTTTTTGATTGGGGTGATCACGGAGTAAAATGAAACCTCCACGCCGAAAGGGTTAACACCCTGAGCAAAAAGCCACAACTTTAAGCACTAAAAAATTAACGTAACTTGACCCAATATTTTGATCAACGAACCAAGTTACCCTAGGGATAACAGCGCAATCCACTTCAAGAGCTCCTATCGACAAGTGGGTTTACGACCTCGATGTTGGATCAGGGTATCCTAGTGGTGCAGCCGCTACTAATGGTTCGTTTGTTCAACGATTAAAACCCTACGTG |
| *4* | *Boana lanciformis* | TWQ_1743 | 2020 | AAAAACATCGCCTCTTGACAAAATATAAGAGGTCCAGCCTGCCCAGTGACCTAGTTCAACGGCCGCGGTATCCTAACCGTGCGAAGGTAGCGTAATCACTTGTTCTTTAAATGAGGACTAGTATGAATGGCACCACGAGGGTTAAGCTGTCTCCTTTTTCTAATCAGTGAAACTAATCTCCCCGTGAAGAAGCGGGGATTATATTATAAGACGAGAAGACCCTATGGAGCTTAAAACAAATTAACACTTGCTTCTGCCTGATTCACTTCATGAACTGACACATAAAGTATAGCACCACTGATTAATAGTTTTCGATTGGGGTGATCATGGAGTAAAAAATAACCTCCACGACGAAAAGGGGCACCTCCCTGAGCCAAAAGCCACAACTTTAAGCACTAAAAAATTGACGCACCTTGACCCAAATTTTTGAGCAACGAACCAAGTTACCCTAGGGATAACAGCGCAATCCACTTCAAGAGCCCCTATCGACAAGTGGGTTTACGACCTCGATGTTGGATCAGGGTGTCCCAGTGGTGCAGCCGCTACTAAAGGTTCGTTTGTTCAACGATTAAAACCCTACGTGA |
| *5* | *Boana punctata* | TWQ_0710 | 2020 | CGCCTCTTGACTAGTTATAAGAGGTATAGCCTGCCCAGTGACACATGTTTAACGGCCGCGGTATCCTAACCGTGCAAAGGTAGCGTAATCACTTGTTCTTTAAATGAGGACTAGTATGAACGGCACCACGAAGGTTATACTGTCTCCTTTCTTTAATCAGTAAAACTAATCTCCCCGTGAAGAAGCGAGGATAAATTTATAAGACGAGAAGACCCTATGGAGCTTTAAACTAACCAGCATTTGCTTTATATAAATTTTTAGCAACTTTAAAGACATCTAGCCCTCTGACTGGTAGTTTTCGATTGGGGTGATCACGGAACAAAAAATAACCTCCGTGTCGAAAGGATATACCTGAGCAAAAAGCGACAGCTTTCCGCACTAAAAAATTAACGTTCATTGACCCAATATTTTGATCAACGAACCAAGTTACCCTAGGGATAACAGCGCAATCTACTTCAAGAGTCCCTATCGACAAGTAGGTTTACGACCTCGATGTTGGATCAGGGTATCCCAGTGGTGCAGCCGCTACTAAAGGTTCGTTTGTTCAACGATTAAAACCCTA |
| *6* | *Cochranella resplendens* | TWQ_035 | 2020 | AACATCGCCTCTTGCCTAAATATAAGAGGTCCAGCCTGCCCAGTGACTCTGTTCAACGGCCGCGGTATCCTAACCGTGCGAAGGTAGCGTAATCACTTGTTCTTTAAATGAGGACTGGTATGAATGGCACCACGAAGGTTATACTGTCTCCTCTTTTTAATCAGTGAAACTAATCTCCCCGTGAAGAAGCGGGGATAAACATATAAGACGAGAAGACCCTATGGAGCTTTAAACTATGTAACACTTGTACTAACCCTAATACAAATTCCAGAAAAATCCTCTACATCTATACAAGATTGTTGCCAGTTTTAGGTTGGGGTGACCACGGAGCAAAAAACAACCTCCACATTGAAAGGAGTTTATTCCTTAACCAAGAGCCACACCTCCACGTATCATTACAATGACATTAATTGACCCAATATATTGATCAACGAACCAAGTTACCCTAGGGATAACAGCGCAATCCACTTCAAGAGCTCCTATCGACAAGTGGGTTTACGACCTCGATGTTGGATCAGGGTATCCCAGTGGTGCAGCCGCTACTAAAGGTTCGTTTGTTCAACGATTAAAACCCTAC |
| *7* | *Dendropsophus bifurcus* | TWQ_1113 | 2020 | AAAAACATCGCCTCTTGCACAATATAAGAGGTCCAGCCTGCCCAGTGACTTAGTTCAACGGCCGCGGTATCCTAACCGTGCGAAGGTAGCGTAATCACTTGTTCTTTAAATGAGGACTAGTATGAATGGCACCACGAGGGTTACACTGTCTCCTTTTTCCAATCAATAAAACTGATCTTCCCGTGAAGAAGCGGGAATTATAATATTAGACGAGAAGACCCTATGGAGCTTCAAACTATATAGCAYTGACTTTTACACTTTACTCTTCWGAGTCCTACACTTATTCTGCTCTTTTTGACTATTAGTTTTGGGTTGGGGTGACCGCGGAGCAAAGATTAACCTCCATGATGAACAAAACCGCTTTTGAACCATAAGCCACAACTTTAAGCACCAATAAATTGACATAAATTGACCCATTTTTTGACCAACGAACCAAGTTACCCTAGGGATAACAGCGCAATCTACTTCAAGAGCCCCTATCGACAAGTGGGTTTACGACCTCGATGTTGGATCAGGGTATCCTAGTGGTGCAGCCGCTACTAAAGGTTCGTTTGTTCAACGATTAAAACCCTACGTGATCT |
| *8* | *Dendropsophus parviceps* | TWQ_0304 | 2020 | TTGCCTAAATATAAGAGGTCTAGCCTGCCCAGTGACTTAGTTCAACGGCCGCGGTATCCTAACCGTGCGAAGGTAGCGTAATCACTTGTTCTTTAAATGGGGACTAGTATGAATGGCATCACGAGGGTTATGCTGTCTCCTTTTTCTAATCAATGAAACTGATCTCCCCGTGAAGAAGCGGGCATAATAATATAAGACGAGAAGACCCTATGGAGCTTTAAACGATTATAGCACTGGCCCACTCATTCCATGTTTCAGAACTACAACATTCACTATAGCCTTAATGACTATTAGTTTTAGGTTGGGGTGACCGCGGAGCAAAAATTAACCTCCACATTGRAAAGAACCTTTCTTGAGCCCAAAGCCACAACTTCAAGCACCAATAAACTGACATACATTGACCCAAGAAATTGATCAACGAACCAAGTTACCCTAGGGATAACAGCGCAATCCACTTCAAGAGCCCCTATCGACAAGTGGGTTTACGACCTCGATGTTGGATCAGGGTATCCCAGTGGCGCAGCCGCTACTAACGGTTCGTTTGTTCAACGATTAAAACCCT |
| *9* | *Dendropsophus reticulatus* | TWQ_2131 | 2020 | AAAAACATCGCCTCTTGCATGATATAAGAGGTCTAGCCTGCCCAGTGACCCAGTTCAACGGCCGCGGTATCCTAACCGTGCGAAGGTAGCGTAATCACTTGTTCTTTAAATGAGGACTAGTATGAATGGCATCACGAGGGTTACACTGTCTCCTTTTTCTAATCAATGAAACTAATCTCCCCGTGAAGAAGCGGGGATATGTACATAAGACGAGAAGACCCTATGGAGCTTCAAACTATTTAGCACTGGCTTTTATATATCCCTACTTCAGAGTTTTTACACTTATTCTAGCCTCTTGACTAATAGTTTTAGGTTGGGGTGACCACGGAGCAAAAATTAACCTCCACGATGAACAGAGCTACTCTTGAACCAAAAGCCACAACTTTAAGCACCAATAAACTGACACAAATTGACCCAATATTTTGAACAACGAACCAAGTTACCCTAGGGATAACAGCGCAATCCACCTTAAGAGCCCCTATCGACAGGTGGGTTTACGACCTCGATGTTGGATCAGGATATCCCAGTGGTGCAGCCGCTACTAATGGTTCGTTTGTTCAACGATTAAAACCCTACGTGATCTG |
| *10* | *Dendropsophus rhodopeplus* | TWQ_0625 | 2020 | ATCGCCTCTTGACCCTTTATAAGAGGTCCAGCCTGCCCAGTGACTAAGTTCAACGGCCGCGGTATCCTAACCGTGCGAAGGTAGCGTAATCACTTGTTCTTTAAATAAGGACTAGTATGAATGGCACCACGAGGGTTATACTGTCTCCTTTTTCTAATCAATGAAACTGATCTCCCCGTGAAGAAGCGGGGATAAATATATAAGACGAGAAGACCCTATGGAGCTTCAAATGACATAGCACTGGCCCTTTCACTTCACATTTCAAAAACACTATATTTAAACAAGCCAAAATGACTATTTATTTTAGGTTGGGGTGACCACGGAGCAAAAACTAACCTCCATGCTGAACGGAGCTACTCCTTAGCTAAAAGCTACAGCTTTAAGCAACAACAAACTGACACCAATTGACCCAATACTTTGATCAACGAACCAAGTTACCCTAGGGATAACAGCGCAATCCATTTCAAGAGCTCCTATCGACAAGTGGGTTTACGACCTCGATGTTGGATCAGGGTATCCCAGTGGTGCAGCCGCTACTAAAGGTTCGTTTGTTCAACGATTAAAACCCT |
| *11* | *Dendropsophus sarayacuensis* | TWQ_0102 | 2020 | TCGCCTCTTGAGCCCTATAAGAGGTCTAGCCTGCCCAGTGACTTAGTTTAACGGCCGCGGTATCCTAACCGTGCGAAGGTAGCGTAATCACTTGTTCTTTAAATGAGGACTAGTATGAATGGCATCACGAGGGTTCCACTGTCTCCTTTTTCCAATCAATGAAACTGATCTTCCCGTGAAGAAGCGGGAATCTTAATATTAGACGAGAAGACCCTATGGAGCTTCAAACTAAATAGCACTGGCTTTTCCCCTATATACCTCAAGGGGTATTAACCCTTATTCTAGCCTTTTAACTATTAGTTTTAGGTTGGGGTGACCGCGGAGCAAAAATTAACCTCCACAATGAATAGCACTATTCTTGAGCCAAAAGCCACAACTTTAAGCACTAACAAACTAACATAAATTGACCCAATATTTTGAACAACGAACCAAGTTACCCTAGGGATAACAGCGCAATCCTCTTCAAGAGCCCTTATCGACAAGTGGGTTTACGACCTCGATGTTGGATCAGGATATCCCAGTGGTGCAGCCGCTACTAAAGGTTCGTTTGTTCAACGATTAAAATCCTACGT |
| *12* | *Leptodactylus wagneri* | TWQ_2940 | 2020 | GGAACCCATAAGAGGTCCAGCCTGCCCAGTGACCCTGTTCAACGGCCGCGGTATCCTAACCGTGCGAAGGTAGCGTAATCACTTGTTCTTTAAATGAGGACTAGTATGAATGGCACCACGAAGGTTATACTGTCTCCTTCCTCTAATCAGTGAAACTAATCTCCCCGTGAAGAAGCGGGGATAAGCCTATAAGACGAGAAGACCCTATGGAGCTTTAAACACAGTAATAAATGCCCCCCCCCCTTTTTCTATTCAAATCTCCGGAAAACTACTTTATCTGAGCATCCTATTTACAAGTTTTAGGTTGGGGTGACCGCGGAGTAAAAAATAACCTCCACAGTGAATGGAATCTTTTTCCTAAACTCAGGGCCACAACCCTAAAAATCAATAAATTGACACCCATTGACCCAATATTTTGATCAATGAACCAAGTTACCCTAGGGATAACAGCGCAATCCACTTCAAGAGCTCCTATCGACAAGTGGGTTTACGACCTCGATGTTGGATCAGGGTACCCCAGTGGTGCAGCCGCTGCTAAAGGTTCGT |
| *13* | *Oreobates quixensis* | TWQ_037 | 2020 | AAAACATCGCCTCTTGTTCTAAATAAGAGGTCCAGCCTGCCCAGTGACTTGTTTAACGGCCGCGGTATCCTAACCGTGCGAAGGTAGCGTAATCACTTGTTTTTTAAATAAAGACCTGTATGAAAGGCACCACGAGAGCCAAACTGTCTCCTTTTTCCTATCAGTGAAACTAATCTTCCCGTGAAGAAGCGGGAATTTTTTAATAAGACGAAAAGACCCTATGGAGCTTTAAACTTCACAACACCTGTTTCTCAATCTTTAATATTCTCTCTAAACACCCTGTTTATGAACTTTCGGTTGGGGCGACCACGGAGAATAACTCACCCTCCGTAATGAGTAACAAAAAGCAACAACTTTATTCATCAGTAAAACTGACATCCATTGACCCAAATCTTTGATCAACGAACCAAGTTACCCTAGGGATAACAGCGCTATCCACTTTAAGAGCCCCTATCGACAAGTGGGCTTACGACCTCGATGTTGGATCAGGGTATCCTGGTGGTGCAGCCGCTACTAACGGTTCGTTTGTTCAACGA |
| *14* | *Pristimantis malkini* | TWQ_016 | 2020 | AACATCGCCTCTTGACTCTTATAAGAGGTCTAGCCTGCCCAGTGACCTAGTTCAACGGCCGCGGTATCCTAACCGTGCGAAGGTAGCGTAATCACTTGTCTTCTAAATAAAGACTAGTATGAAAGGCACCACGAGAACTAAACTGTCTCCCTTTTCCAATCAGTGAACCTAATGCCCCCGTGAAGAAACGGGGATTTTACAATAAGACGAGAAGACCCTATGGAGCTTTAAACATCATAATCACCTGCCTACTAAACCAAGATACTCAGGTCCCCCACCCAAACCCATGATGATTATATGTTTTTGGTTGGGGTGACCGCGGAGAAAAACACATCCCCCGCGATGAAAGCCCACCCAAACAACAAGCCACAGCTTTATGTATCAACAAATTGACACTATTGACCCAATATATTGATTAACGAACCAAGTTACCCTAGGGATAACAGCGCAATCCACTTCAAGAGCCCCTATCGACAAGTGGGCTTACGACCTCGATGTTGGATCAGGGTATCCTAGTGGTGCAGCCGCTAC |
| *15* | *Pristimantis variabilis* | TWQ_018 | 2020 | TCGCCTCCTGTTCTCATAGGAGGTCCAGCCTGCCCAGTGACTTAATTTAACGGCCGCGGTATCCTAACCGTGCGAAGGTAGCGTAATCACTTGTCTTCTAAATAAAGACTTGTATGAAAGGCATCACGAGGGCTTAACTGTCTCCTTTCTCCAATCAGTGAACCTAATCTCCCCGTGAAGAAGCGGGGATAACTATATTAGACGAGAAGACCCTATGGAGCTTTAAATACTATAACATATGCTTTCCCCCCTATATCACCGAGGTGCCTCACCTTTCACAGCTAATTGATTATATATTTTTGGTTGGGGTGACCGCGGAGAATAAACCATCCTCCACAATGAATGGGGATCCCCCCCAATAAACAAGCCACAACTTTACCCATCAATAAATTGACTCTCATGACCCAATATTTGATCAACGAACCAAGTTACCCTAGGGATAACAGCGCAATCCACTTTAAGAGCTCCTATCGACAAGTGGGCTTACGACCTCGATGTTGGATCAGGGTATCCTAGT |
| *16* | *Rhaebo ecuadorensis* | TWQ_3639 | 2020 | ACATCGCCTCTTGATTAGTTATAAGAGGTCCAGCCTGCCCAGTGACCCTGTTCAACGGCCGCGGTATCCTAACCGTGCGAAGGTAGCGTAATCACTTGTTCTTTAAATAAGGACTAGTATGAATGGCACCACGAGGGCTACACTGTCTCCTTTTTCTAATCAGTGAACCTAATCTTCCCGTGAAGAAGCGGGAATAAACCTATAAGACGAGAAGACCCTATGGAGCTTTAAACAATATAGCATTTGCCATATAACATAAAAATTTCCGAATATTCACTTCTTAAAGCAGTATGACTATAAGTTTTTGGTTGGGGTGACCGCGGAGCATAACACAACCTCCATGTTGAATGAAGATTATTCTAAGCCGCGAACCACACTTCTAAGCATCAAAATACTGACACCAATTGACCCAATATATTTGAACAACGAACCAAGTTACCCTAGGGATAACAGCGCAATCCACTTCAAGAGCTCCTATCGACAAGTGGGTTTACGACCTCGATGTTGGATCAGGGTCTCCCAGTGGTGTAGCCGCTACTAAAGGTTCGTTTGTTCAACGATTAAAACCCTACGTGATC |
| *17* | *Rhinella marina* | TWQ_2728 | 2020 | CATCGCCTCTTGACTCTTATAAGAGGTCCAGCCTGCCCAGTGACCATGTTCAACGGCCGCGGTATCCTAACCGTGCGAAGGTAGCGTAATCACTTGTTCTTTAAATRCGGACTAGTATGAATGGCACCACGAAGGTTATACTGTCTCCTTTTTCTAATCAGTGAAACTAATCTCCCCGTGAAGAAGCGAGGATAYACCTATAAGACGAGAAGACCCTATGGAGCTTTAAACAACACAGCAATTACCTGCTAAACTAAGAAGTTTCTGAACATTTTCAATCTTTTAAGTAATCTGACTGCAAGTTTTTGGTTGGGGTGACCACGGAGCATAACACAACCTCCAYGTTGAAAGAATTCTTTCTAAGCCAAGAACAACCTTTCTAAGCATCAATATATTGACATCCATTGACCCAATACATTTGACCAACGAACCAAGTTACCCTAGGGATAACAGCGCAATCCACTTCAAGAGCTCCTATCGACAAGTGGGTTTACGACCTCGATGTTGGATCAGGGTA |
| *18* | *Scinax aff cruentomma* | TWQ_012 | 2020 | CGTAATCACTTGTTCTTTAAATGGGGACTAGTATGAATGGCACCACGAGGGTTATACTGTCTCCTTTCTCTGATCAGTGAAACTAATCTCCCCGTGAAGAAGCGGGGATACAGCTATAAGACGAGAAGACCCTATGGAGCTTTAAATCTCTTTTCAAACGCTAAAATACTTATACAAGTCCCAGACTTACCCTCACACTTACCTAGCGCCATGAAAATGATTTTAGGTTGGGGTGACCGCGGAGCACAAAAAAACCTCCACGCTGAACTAGACGTCCAAACCTTGAGCTAAGGGCTACGACCCGATGCACTAAAAAATTAACACCAATTGACCCAATATTTATTTGATCAACGAACCAAGTTACCCTAGGGATAACAGCGCAATCCATTTCAAGAGTCCATATCGACAAATGGGTTTACGACCTCGATGTTGGATCAGGGTCTCCCAGTGGTGCAGCCGCTACTAATGGTTCGTTTGTTCAACGATTAAAACCCTA |
| *19* | *Scinax garbei* | TWQ_008 | 2020 | AAAAACATCGCCTCTTGCCTAAAATAAGAGGTCCAGCCTGCCCAGTGACACATGTTCAACGGCCGCGGTATCCTAACCGTGCGAAGGTAGCGTAATCACTTGTTCTTTAAATGAGGACTAGTATGAATGGCATCACGAGGGTTATACTGTCTCCTTTTTCTAATCAGTGAAACTAATCTCCCCGTGAAGAAGCGGGGTTGAAATTATAAGACGAGAAGACCCTATGGAGCTTTAAATTTCCACTCAAATGCTTATTAACCTTTATACTTCTTAGTATTATTAAAAAGTACCTTAGCATTATGAGCCAAAATTTTAGGTTGGGGTGACCGCGGAGTACAAAATAACCTCCACATTGAAACAGGCTTACAAACCCTGAGCAACGGACAACAATCCTATGCACTAAAAAACTAACATCCATTGACCCAAGTTTTTGATCAACGAACCAAGTTACCCTAGGGATAACAGCGCAATCCTCTTCAAGAGTTCCTATCGCCAAGAGGGTTTACGACCTCGATGTTGGATCAGGGTATCCCAGTGGTGCAGCCGCTACTAATGGTTCGTTTGTTCAACGATTAAAACCCTACGTGATCT |
| *20* | *Scinax ruber* | TWQ_2022 | 2020 | AAAACATCGCCTCTTGAACAAAAATAAGAGGTCCAGCCTGCCCAGTGACTAATGTTCAACGGCCGCGGTATCCTAACCGTGCGAAGGTAGCGTAATCACTTGTTCTTTAAATGAGGACTAGTATGAATGGCACCACGAGGGTTATACTGTCTCCTCTTTCTAATCAGTGAAACTAATCCCCCCGTGAAGAAGCGGGGATTAAACTATAAGACGAGAAGACCCTATGGAGCTTTAAATTTACCCTCAAACGCCAAAATCATACAGCAAGTTTTAAACTTCACCACACGAACTTTCGCGCCATGAGGGCAACTTTTAGGTTGGGGTGACCATGGAGTATAAAATAACCTCCATACTGAACAGGCCTCCAAGCCTCGAACCAAGAGCCACGACCCGCCGCACTAAAATATTAACATCCATTGATCCAATGTATTTTGATCAACGAACCAAGTTACCCTAGGGATAACAGCGCAATCTACTTCAAGAGTTCCTATCGACAAGTGGGTTTACGACCTCGATGTTGGATCAGGGTGTCCCAGTGGTGCAGCCGCTACTAATGGTTCGTTTGTTCAACGATTAAAACCCTAC |

**Table S4**. **List of the most common vertebrate species in the Tena River microbasin, Napo, Ecuador and their respective sequences. sequences of bacteria sequenced with ONT are included**

| Group | Species | Code | Sequences |
| --- | --- | --- | --- |
| Mammals | *Homo sapiens* | MW698620 | AAGGTAGCATAATCACTTGTTCCTTAAATAGGGACCTGTATGAATGGCTCCACGAGGGTTCAGCTGTCTCTTACTTTTAACCAGTGAAATTGACCTGCCCGTGAAGAGGCGGGCATAACACAGCAAGACGAGAAGACCCTATGGAGCTTTAATTTATTAATGCAAACAGTACCTAACAAACCCACAGGTCCTAAACTACCAAACCTGCATTAAAAATTTCGGTTGGGGCGACCTCGGAGCAGAACCCAACCTCCGAGCAGTACATGCTAAGACTTCACCAGTCAAAGCGAACTACTATACTCAATTGATCCAATAACTTGACCAACGGAACAAGTTACCCTAGGGATAACAGCGCAATCCTATTCTAGAGTCCATATCAACAATAGGGTTTACGACCTCGATGTTGGATCAGGACATCCCAATGGTGCAGCCGCTATTAAAGGTTCGTTTGTTCAACGATTAAAGTCCTACGTGATCTGAGTTCAGACCGGAGTAATCCAGGTCGGTTTCTATCTACTTCAAATTCCTCCCTGTACGAAAGGACAAGAGAAATAAGGCCTACTTCACAAAGCGCCTTCCCCCGTAAATGATATCATCTCAACTTAGTATTATACCCACACCCACCCAAGAACAGGGTTTGTTAAGATGGCAGAGCCCGGTAATCGCATAAAACTTAAAACTTTACAGTCAGAGGTTCAATTCCTCTTCTTAACAACATACCCATGGCCAACCTCCTACTCCTCATTGTACCCATTCTAATCGCAATGGCATTCCTAATGCTTACCGAACGAAAAATTCTAGGCTATATACAACTACGCAAAGGCCCCAACGTTGTAGGCCCCTACGGGCTACTACAACCCTTCGCTGACGCCATAAAACTCTTCACCAAAGAGCCCCTAAAACCCGCCACATCTACCATCACCCTCTACATCACCGCCCCGACCTTAGCTCTCACCATCGCTCTTCTACTATGAACCCCCCTCCCCATACCCAACCCCCTGGTCAACCTCAACCTAGGCCTCCTATTTATTCTAGCCACCTCTAGCCTAGCCGTTTACTCAATCCTCTGATCAGGGTGAGCATCAAACTCAAACTACGCCCTGATCGGCGCACTGCGAGCAGTAGCCCAAACAATCTCATATGAAGTCACCCTAGCCATCATTCTACTATCAACATTACTAATAAGTGGCTCCTTTAACCTCTCCACCCTTATCACAACACAAGAACACCTCTGATTACTCCTGCCATCATGACCCTT |
| Mammals | *Bos taurus* | MW423198 | AGGTCGGTTTCTATCTATTACGTATTTCTCCCAGTACGAAAGGACAAGAGAAATAAGGCCAACTTTAAATCAAGCGCCTTAAGACAACCAATGATAACATCTCAACTGACAACACAAAACCCTGCCCTAGAACAGGGCTTAGTTAAGGTGGCAGAGCCCGGTAATTGCATAAAACTTAAACTTTTATATCCAGAGATTCAAATCCTCTCCTTAACAAAATGTTCATAATTAACATCTTAATACTAATTATTCCCATCCTATTGGCCGTAGCATTCCTTACGTTAGTGGAACGAAAAGTTCTAGGCTATATACAACTCCGAAAAGGTCCAAATGTCGTAGGTCCATATGGCCTACTTCAACCCATCGCCGATGCAATCAAACTTTTCATTAAAGAACCACTACGACCCGCTACATCTTCAGCCTCAATATTTATCCTAGCACCTATCATAGCTTTAGGCCTAGCCTTAACCATGTGAATTCCCCTACCAATACCCTATCCTCTTATCAACATAAACCTAGGAGTCCTATTTATACTAGCCATATCAAGCCTAGCCGTATACTCCATTCTCTGATCAGGCTGAGCTTCCAACTCAAAATACGCACTAATCGGAGCCCTACGAGCAGTAGCACAAACAATCTCATACGAAGTAACGCTAGCAATTATCCTGTTATCAGTACTCCTAATAAGTGGGTCCTTTACCCTCTCCACATTAATTACTACACAAGAACAAATATGGTTAATCCTCCCAGCATGGCCTCTAGCAATAATATGATTTATCTCAACACTAGCAGAAACAAACCGAGCTCCATTTGATTTAACTGAAGGAGAATCAGAGCTAG |
| Mammals | *Oryctolagus cuniculus* | MN953621 | AAGACATCGGCACTCTTTATCTCCTATTTGGAGCTTGAGCTGGGATGGTGGGAACAGCCCTCAGCCTGCTAATTCGAGCAGAATTAGGTCAGCCAGGGACTCTACTCGGGGATGATCAAATCTATAATGTAATCGTCACCGCACATGCCTTTGTAATAATCTTCTTTATAGTCATGCCTATTATAATTGGAGGCTTCGGGAACTGGCTTGTCCCCCTGATAATTGGGGCTCCTGACATAGCCTTCCCCCGAATAAATAATATGAGCTTCTGACTTCTCCCCCCTTCATTCCTTCTTCTACTAGCCTCCTCAATAGTAGAAGCTGGGGCGGGGACTGGCTGAACTGTTTAT  CCACCTCTAGCCGGTAATCTTGCACATGCTGGAGCCTCAGTGGATCTTACTATTTTCTCCCTTCACTTAGCTGGAGTATCATCTATTTTAGGGGCTATTAACTTTATTACAACTATTATTAATATGAAACCCCCTGCAATATCTCAATATCAAACCCCCTTATTCGTATGATCTGTTCTAACCACAGCCGTACTTCTTCTTCTCTCTTTACCAGTCCTAGCTGCTGGCATTACAATGCTTTTAACAGACCGAAACTTAAATACAACCTTCTTTGATCCTG |
| Mammals | *Canis lupus* | MN181404 | ATGAATAATTATTCAAGAATTAACGAAAAATAACATAATTATTATTCCAACACTAATGGCTATCACCGCTCTACTTAACTTATATTTCTACCTGCGACTCACATATAGCACCGCACTTACCATATTTCCATCCACAAACAACATAAAAATAAAATGACAGTTCGAATACACAAAAAAGGCAACCCTATTACCCCCCTTAATTATTACCTCAACTATACTACTCCCACTAACACCTATACTATCAGTCTTGGACTAGGAGTTTAGGTTAGACCAGACCAAGAGCCTTCAAAGCTCTAAGCAAGTGCTACACACTTAACCCCTGATCAAATCACCTCTAAGGGCTGCAAGAATCTATCTTACATCAATTGAATGCAAATCAAACACTTTAATTAAGCTAAGCCCTCCCTAGATTGGTGAGCTTCTACCTCACGAAATTTTAGTTAACAGCTAAATACCCTAGTAACTGGCTTCAATCTAC |
| Mammals | *Peropteryx macrotis* | AY395850 | AATTGAAGTACTAAAAAGCAAAGATTAGACCTTTTACCTTTTGCATAATGAATTAACTAGAAAACCCTTAACAAAGAGAACTGAAGCTAAGCCCCCCGAAACCAGACGAGCTATCTAAAGACAGCCCAAAGGGGCCAACCCGTCTATGTGGCAAAATAGTGAGAAGATCTTTAGATAGAGGCGAAAAACCAACCGAGCCTGGTGATAGCTGGTTGTCCAGTAAATGAATTTTAGTTCACTTTAAGCTTACCCACTGCAATCATAAGCCTCATGTAAGCTTAAAATATAGTCTAAAAGGGTACAGCCTTTTGGACCCAGGATACAACCTTCATTAGAGAGTAAATACCAAAACTACCATAGTTGGCTTAAAAGCAGCCACCCATTGAGAAAGCGTTCAAGCTCAACAATTAGACCATCTTAATTACACACGCACCAAACAACTCCTAATCCAAATACTGGACTGTTCTATTATTAAATAGAAGCAATACTGTTAATATGAGTAATAAGAAAATATTTTCTCCCTGCACAAGCATATATCAGAACGAATAATTCACTGATAGTTAACAAACTTTAAAATTAACCTAAAGATTAACTTCTTAACTCCCAATTTGTTAACCCAACACAGGCGTGCACCACCAAAGGGAAAGATTAAAAGGGATAAAAGGAACTCGGCAAACACAAACCCCGCCTGTTTACCAAAAACATCACCTCTAGCCTTATAAGTATTAGAGGCACTGCCTGCCCAGTGACAACAGTTTAACGGCCGCGGTCCTGACCGTGCAAAGGTAGCATAATCACTTGTTCTCTAAATAGGGACTCGTATGAATGGCCACACGAGGGTTTAACTGTCTCTTATCCCCAATCA  ATGAAATTGACCTTCCCGTGCAGAGGCGGGAATAACCTAATAAGACGAGAAGACCCTATGGAGCTTTAATTAACTAGTCTATAGAAAAATAACACCAGCCCAATAGGAATAACAAGACTCTAACTAGATTAGTAATTTAGGTTGGGGTGACCTCGGAATAAAAAACAACTTCCGAGTGATTAATATCTAGACTTACCAGTCAAAATAAACTATCATCACTTATTGATCCAATCTCTTGATCAACGGACCAAGTTACCCTAGGGATAACAGCGCAATCCTATTTAAGAGTCCATATCGACAATAGGGTTTACGACCTCGATGTTGGATCAGGACATCCTAGTGGTGCAGCCGCTACTAAGGGTTCGTTTGTTCAACGATTAACAGTCCTACGTGATCTGAGTTCAGACCGGAGTAATCCAGGTCGGTTTCTATCTATTTTATGCCTCCCCCAGTACGAAAGGACAAGAGAGGCGGGGCCCACCTCATCACGCGCCCCCAAACCAAACAGATGCTGTAATATAAATCTGAAAGGATATAAAATATCAACCCTAGACCAGGG |
| Fish | *Oreochromis niloticus* | KM434157 | TGTAATTGTTACAGCACATGCTTTTGTAATAATTTTCTTTATAGTAATGCCAATTATGATTGGAGGCTTTGGAAACTGACTAGTACCACTCATGATTGGTGCCCCAGATATGGCCTTCCCTCGAATGAACAACATGAGTTTCTGACTCCTCCCTCCCTCATTCCTCCTCCTCCTCGCCTCATCTGGAGTCGAAGCAGGTGCCGGCACAGGGTGAACTGTTTACCCCCCGCTCGCAGGCAATCTTGCCCATGCTGGGCCTTCTGTCGACTTAACCATCTTCTCCCTCCACTTGGCCGGGGTGTCATCTATTCTAGGCGCAATTAATTTCATTACAACAATCATTAACATGAAACCCCCCGCCATCTCTCAATATCAAACACCCCTATTTGTATGGTCCGTTCTAATTACCGCAGTATTACTTCTTCTATCCCTACCCGTTCTTGCCGCCGGCATCACAATACTTCTCACAGACCGAAACCTAAACACAACCTTCTTTGATCCTGCCGGAGGAGGAGACCCCATCCTTTACCAACACTTATTCTGATTCTTTGGACACCCTGAAGTTTACATTCTTATCCTCCCCGGCTTTGGAATAATCTCCCACATTGTTGCTTACTATGCGGGTAAAAAAGAACCTTTCGGATATATGGGAATGGTCTGGGCCATGATGGCTATCGGCCTCCTAGGGTTCATTGTATGAGCCCATCACATGTTCACCGTAGGGATGGACGTAGACACACGGGCTTACTTTACTTCCGCCACAATAATTATTGCCATCCCAACCGGAGTAAAAGTCTTCAGCTGACTGGCCACTCTGCACGGCGGTGCCATTAAATGAGAAACCCCACTCTTATGAGCGCTAGGTTTCATCTTCCTCTTTACAGTTGGAGGTCTAACCGGAATTGTCCTA |
| Fish | *Synbranchus marmoratus* | KC880298 | CAAAAACATCGCCTCTTGCTCCCCTAATATAAGAGGTCCCGCCTGCCCCCTGACTATACGTTTAATGGCCACGGTATCCTAACCGTGCAAAGGTAGCGCAATCATTTGTCTTCTAATTAAAGACCTGTATGAACGGCATAACGAGAGCTTACCTGTCTCCTCTTTAAAGTCAATAAAATTGATCTCCCCGTGCAGAAGCGAGGATATATACATAAGACGAGAAGACCCTGTGGAGCTTTAGGCACTAAAATATGTGTACATTACACAATTACACCCTTATTTCAATGCCTTCGGTTGGGGCGACCAAGGAGAATTAATCAACCCCCACGTGGAATAGAAGTACTCCTTCTCAAACCAAGAGTTACAACTCTAATTAATAGAATTTCTAACCATTCAACATAAGACCCGGCAACGCCGATCTACGAACCAAGTTACCCCAGGGATAACAGCGCAATCCCCTTTAAGAGCCCATATCGACAAGGGGGTTTACGACCTCGATGTTGGATCAGGACACCCCAATGGTGCATCCGTTATTAAAGGTTCGTTTGTTCAACGATTAAAGTCCTACGTGATC |
| Fish | *Chaetostoma microps* | KP959836 | CAAAAACATCGCCTCCCGCAAAAATCAATATATAGGAGGTCTTGCCTGCCCAGTGACTATAAGTTAAACGGCCGCGGTATTTTGACCGTGCAAAGGTAGCGCAATCACTTGTCTTTTAAATGAAGACCTGTATGAATGGTGAAACGAGGGCTTAACTGTCTCCCCACTCTAGTCAATGAAATTGATCTACCCGTGCAGAAGCGGGTATACTAATACAAGACGAGAAGACCCTTTGGAGCTTAAGACAAAAAACCAACTACGTCAAAAGTCCTAAAAACTAAACCAAGTAGCACTGGTTTCAATCTTCGGTTGGGGCGACCGCGGGAGAAAGCAAAGCTCCCATGCGGACTGGGCTAAACCCCCTAAAACTAAGAGAGACATCTCCAAGGCACAGAACATCTGACCAAAAAGATCCGGCTTCATGCCGACCAACGGACCAAGTTACCCTAGGGATAACAGCGCAATCCCCTTCCAGAGTCCATATCGACAAGGGGGTTTACGACCTCGATGTTGGATCAGGACATCCTAATGGTGCAGCCGCTATTAAGGGTTC |
| Fish | *Astroblepus sp.* | HM049031 | CGCCTCCTGCAAAACTCAACATATAGGAGGTCTTACCTGCCCAGTGACTACAAGTTAAACGGCCGCGGTATTCTGACCGTGCAAAGGTAGCGCAATCACTTGTCTTTTAAATGAAGACCTGTATGAATGGTTAAACGAGGGCCTAACTGTCTCCCCATTCCAGTCAATGAAATTGATCTGCCCGTGCAGAAGCGGACATAAATATACAAGACGAGAAGACCCTTTGGAGCTTAAGATAAAAGACCAATTATGTCAATAACCCTACAAGAAAATAAACTAAATAACCGCTGGTCCAAATCTTCGGTTGGGGCGACCACGGGAAAAAACAAAACTCCCATGTAGACAGGGAAAATCCCCTAAAACTAAGAGAGACATCTCTAAGGAACAGAACTTCTGACTATAAAGACCCGGCCATGAGCCGACCAACGAACCAAGTTACCCAAGGGATAACAGCGCAATCCCCTTCCAGAGTCCATATCGACAAGGGGGTTTACGACCTCGATGTTGGATCAGGACATCCTAATGGTGCAGCCGCTATTAAGGGTTCGTTTGTTCAACGATTAAAGTCCT |
| Fish | *Colossoma macropomum* | HQ171343 | CAAAAACATCGCCTCCTGCAAAAATCAACGTATAGGAGGTCCTGCCTGCCCAGTGACAACCAGTTAAACGGCCGCGGTATTTTGACCGTGCTAAGGTAGCGCAATCACTTGTCTTTTAAATGAGGACCTGTATGAATGGCGGAACGAGGGCTTAACTGTCTCCTTTTTCTAGTCAATGAAATTGATCTACCCGTGCAGAAGCGGGTATACTAATACAAGACGAGAAGACCCTTTGGAGCTTAAGATAAAAGGCCAACTATGTCAAAGGCCCAAATAAAACTAAACCTAACAAAACAGTTAACTGGCCAATATCTTCGGTTGGGGCGACCGCGGGGGAAAACAAAGCCCCCACGTGGAATGGGGAATACTCCCTAAAACCAAGAGAGACATCTCTAAGTCACAGAACATCTGACCAATAGATCCGGCCAATCAAGCCGATCAACGGACCAAGTTACCCTAGGGATAACAGCGCAATCCCCTCCAAGAGTTCATATCGACAAGGGGGTTTACGACCTCGATGTTGGATCAGGACATCCTAATGGTGCAGCCGCTATTAAGGGTTCGTTTGTTCAACGATTAAAGTCCTACGTGATCTGAG |
| Bird | *Gallus gallus* | LC082227 | CGGCACATGGGCGGGCATAGCCGGCACAGCACTTAGCCTTCTAATCCGCGCAGAACTAGGACAGCCCGGAACTCTCTTAGGAGACGACCAAATTTACAATGTAATCGTCACAGCCCATGCTTTCGTCATAATCTTCTTTATAGTTATACCCATCATGATCGGTGGCTTCGGAAACTGACTAGTCCCGCTTATAATCGGTGCCCCAGACATAGCATTCCCCCGCATAAATAACATAAGCTTCTGACTCCTCCCTCCCTCCTTCCTTCTCCTACTAGCCTCATCTACCGTAGAAGCTGGGGCCGGCACAGGATGGACAGTTTACCCCCCTTTAGCCGGCAACCTAGCCCACGCTGGCGCATCAGTAGACCTAGCCATCTTTTCATTACACTTAGCAGGTGTTTCCTCCATTCTAGGAGCCATCAACTTTATCACTACCATCATCAACATAAAACCCCCCGCACTGTCACAATACCAAACACCCCTATTCGTATGATCCGTCCTCATTACTGCCATCCTACTACTCCTCTCCTTACCCGTCCTAGCAGCTGGGATTACCATACTACTTACCGACCGCAACCTTAACACCACATTCTTCGACCCAGCTGGAGGAGGAGACCCAATCCTATACCAACACCTATTCTGATTCTTCGGTCACCCCGAAGTTTACATCCTCATCCTCCCAGGTTTCGGAATAATTTCC |
| Bird | *Coragyps atratus* | MN720440 | CTCCAGCACTATAGTTGTTGCCGGAATCTTCCTACTTATCCGCACCTACCCCATACTTTCCAGCAACCAAACTGCTCTCACCTCCTGCCTATGCCTAGGAGCCCTGTCCACACTATTTGCCGCCACATGTGCACTCACACAAAACGACATCAAAAAAATCATTGCCTTCTCAACCTCTAGCCAACTGGGCCTAATAATAGTTACTATTGGGCTAAACCTCCCCCAGCTAGCCTTCCTTCACATCTCGATACATGCCTTCTTCAAAGCTATACTATTCCTCTGCTCAGGCTCAATCATCCATAACCTCAACGGAGAACAAGATATCCGAAAGATGGGAGGACTACAAAAAACACTCCCAACAACCACCTCATGCCTAACCATCGGCAACCTAGCCCTAACTGGAACTCCATTCCTAGCAGGTTCTACTCAAAAGACCTCATCATTGAAAGCCTAAACACCTCCTATCTAAACACCTGAGCACTACTACTAACACTCTTAGCTACCGCATTCACAGCAACCTACAGCCTACGTATAACCTTACTAGTCCAAACAGGACACACCCGAACCCCAACAATCACCCCCATAAACGAAAATAACCCAACAATCATTAACCCAATCACCCGCCTCGCTCTAGGTAGCATCCTGGCCGGTCTACTCATCACATCATATACTCTCCCCTCAAAAACACCCCCCATAACCATGCCCATACTCACAAAAACTGCAGCAATCATCGCCACAATCTTGGGGGCAATCTTAGCCCTAGAACTTCTAAACATAACACAC |
| Bird | *Brotogeris cyanoptera* | HM627323 | TTCCTCCCCCCTACTAGTCTTAACCTGCTGGCTTACGCCACTCATAATTATTGCAAGCCAAAACCACCTACAGCAGGAACCACCCACACGAAAACGAATCTTCACAACAACCCTAATCACAGTACAACCCCTCATCATCCTAGCCTTCTCAACCACAGAGCTCATAATATTTTATATCTTCTTCGAAGCAACCCTAATCCCAACACTAATCCTAATCACCCGATGAGGAAGTCAGCCAGAACGCCTAGGAGCCGGAATCTACCTCCTCTTCTACACACTCATCAGCTCCCTCCCCCTACTAATCGCAATCCTGTACATACACTCACAGACAGGAACCCTACTCTTCCCCACCCTAAAGCTACTTCCCTACCTCCCATCAACCACACCAGCCAAATACTGATCCGCCCTCCTCCTCAACCTGCCCTCCTCACAGCCTTCATAGTAAAAGCCCCACTATACGGACTTCACCTCTGACTCCCAAAAGCCCACTAGAGGCTCCAATCGCAGGATCAATACTACTCGCCGCCCTTCTCCTCAAACTAGGCGGATATGGCATCATACGCATTACCTGCCTAACAAACCCCCCCACAAACAGCCTCCTCCACTACCCATTCATTACCCTCGCCCTATGAGGCGCACTAATAACCAGCTCAATCTGCCTCCGCCAAATCGACCTAAAATCACTCATCGCCTACTCC |
| Bird | *Anas platyrhynchos domesticus* | MK770342 | GCAGCCATAGCAATCGCCATGCTATCCCTACTTAGCCTATTCTTCTACCTACGCCTCGCATACCACTCAACAATCACCCTCCCACCAAACTCGTCCAACCACATAAAACAGTGGTACACTAGCAAACCCCCAAGCACGCCCACCGCAATCCTAGCCTCACTATCAATCCTCCTACTCCCCCTCTCCCCCATAGTCCACGCTATTGTCTAGAAACTTAGGATAACACCCACCTAAACCGAAGGCCTTCAAAGCCTTAAATAAGAGTTAAACCCTCTTAGTTTCTGCGCTAAGACCAACAGGACACTAACCTGTATCTCATGGATGCAAACCAGACGCTTTAATTAAGCTAAAGCCTTTACTAGACAGACGGGCTTCGATCCCGCAAAATTTTAGTTAACAGCTAAACGCCCAAACCTACTGGCCTCTGCCTAAGGCCCCGGTACACTCTCGTGCACATCGATGAGCTTGCAACTCAACATGAACTTCACTACAGGGCCGATAAGAAGAGGAATTGAACCTCTGTAAAAAGGACTACAGCCTAACGCTTTAAACACTCAGCCATCTTACCCGTGACCTTCATCAATCGATGACTATTTTCTACCAATCACAAAGACATCGGTACTCTATACCTTATCTTCGGGGAATGAGCCGGAATAATTGGCACAGCACTCAGCCTACTGATCCGGGCAGAACTAGGCCAGCCAGGGACCCTCCTGGGCGACGACCAAATTTATAACGTGATCGTCACCGCTCACGCCTTCGTAATAATCTTCTTCATGGTAATGCCCATCATAATTGGAGGGTTCGGCAACTGATTGGTCCCCCTGATAATCGGTGCCCCCGACATAGCATTCCCACGAATAAACAACATAAGCTTCTGACTCCTCCCACCATCATTCCTCCTTCTACT  CGCCTCATCCACTGTAGAAGCTGGCGCTGGTACGGGTTGAACCGTATACCCACCTCTAGCAGGCAACCTAGCCCACGCCGGAGCCTCAGTGGACCTGGCTATCTTCTCACTTCACCTGGCTGGTGTCTCCTCCATCCTCGGAGCCATTAACTTCATTACCACAGCCATCAACATAAAACCCCCCGCACTCTCACAATACCAAACCCCACTTTTCGTCTGATCAGTCCTAATTACCGCCATCCTGCTCCTCCTATCACTCCCCGTCCTCGCCGCCGGCATCACAATGCTACTAACCGACCGAAACCTAAACACCACATTCTTTGATCCTGCCGGAGGGGGAGACCCAAT |
| Bird | *Pteroglossus inscriptus* | GQ422994 | GCCTGTGAAATGAACCTCAGTTCACCCCTGACCTCTCCTCACGCCCACCAACCCAAGCCCCAATGTAGACGAACAGGAGTTACTTAAAGGAGGTACAGCCCCTTTAAAAAAGGATACAACCTCTTACAGCGGATAACCCACCCCATAAACCCAACTGTAGGCCTTCAAGCAGCCACCAACAAAGAGTGCGTCAAAGCTCCTCAATAAAAAACACTCAAACCACGCGAATCCCTCCTCACTAACAGGCCAACCTATCCCCATAGGAGAATTAATGCTGAAATAAGTAACTGGGGCCCTCCCCCTCTCAAGCGCAAACTTACATCCTCCATTATTATCCAAAACACGATACCCACACCTCAACAAGCACGCTCTATCAACGAACCCTGTTACACCAACCCAGGTGCGCAACAATAGAAAGACCCAAATCTGTAGAAGGAACTAGGCAAACCCAAGGCCCGACTGTTTACCAAAAACATAGCCTTCAGCACACCAAGTATTGAAGGTGATGCCTGCCCAGTGACCTCACCACGTTCAACGGCCGCGGTATCCTA |
| Reptile | *Imantodes cenchoa* | MH140783 | AACCTTTAGCCAGAACAAATATTAAAGGCAACGCCTGCCCAGTGAACAATTAAACGGCCGCGGTATCCTAACCGTGCAAAGGTAGCGTAATCACTTGTCTATTAATTGTAGACCCGTATGAAAGGCAAAATGAGAGTCTGCCTGTCTCTTATAATAAATCAATTAAACTGATCTCCTAGTAAAAAAGCTAGAATACCAACATAAGACCAGAAGACCCTGTGAAGCTTAAACTAAACTATTAAACCCTATAATACCTACTTTCGGTTGGGGCGACCTTGGAAAAAAAAAGAACTTCCAAACACATGACCATAACTCATTCACCAGGCCAACAAGCCTAACACGACCCAGTACAACTGACAACTGAAACAAGTTACTCCAGGGATAACAGCGCTATCTTCTTCAAGAGTCCATATCAAAAAGAAGGTTTACGACCTCGATGTTGGATCAGGACATCCTAATGGTGCAGCCGCTATTAAGGGTTCGTTTGTTCAACGATTAACA |
| Reptile | *Leptodeira annulata* | MH140814 | AACAATTAAACGGCCGCGGTACCCTAACCGTGCAAAGGTAGCATAATCATTTGTCTATTAATTGTAGACCAGTATGAAAGGCAAAATGAGGGTCTATCTGTCTCTTATAATGAATCAATTAAACTGATCTCCTAGTAAAAAAGCTAGAATTATAACATAAGACCAGAAGACCCTGTGAAGCTTAAACTAAAATATTAAACCACATAATACATACTTTTGGTTGGGGCGACCTTGGAAAAAAAAAGAACTTCCAAACACATGATCATAACTCATACCAAACATAGGCCCACAAGCCTTTAATTACGACCCAGTATAACTGATAACTGAACAAAGTTACTCCAGGGATAACAGCGCCATCTTCTTCAAGAGTCCATATCAAAAAGAAGGTTTACGACCTCGATGTTGGATCAGGACATCCTAATGGTGCAGCCGCTATTAAGGGTTCGTTTGTTCAACGATTAACAGTCCT |
| Reptile | *Amphisbaena fuliginosa* | MH140476 | AGCCTTTAGCCACCACAAGTATTGAAGGTATCGCCTGCCCAGTGAATCTTTTAACGGCCGCGGTATCCTAACCGTGCAAAGGTAGCGTAATCATTTGTTCTTTAAATAAGGACTTGTATGAATGGCACCATGAGGAACGACTTGTCTCTCGTACTAAGTCTATGAAACTGACCTCCAAGTACAAACGCTTGAATGAACACACAAGACAAGAAGACCCTGTGGAGCTTTAAACCTACCAGCCCACCAATTATCCCACTGAGCTGTGGTTTTTAGTTGGGGCGACTTTGGAGCAAAACAAAACCTCCAAACTACTTATCTTAGGCCATCACGCCGATATGCCAATCAACTAATTGACCCAATAAAATTGATTAATGAACCAAGTTACCCCAGGGATAACAGCGCAATCTTCTTCTAGAGTCCCTATCGACAAGAAGGTTTACGACCTCGATGTTGGATCAGGATACCCAAATAGTGCAGCCGCTATTAAAGGTTCGTTTGTTCAACGATTAATAATCCT |
| Bacteria | *Uncultured Turicimonas sp.* | MN135770 | TAGATATTTGGAAGAACACCGATGGCGAAGGCAGCCCCTGGGACGCAACTGACGCTCATACACGAAAGCGTGGGARCAAACAGGATTAGATACCCTGGTAGTCCACGCCCTAAACGATGTCAACTAGTTGTTGGGAGGTAAAACTTTCAGTAACGCAGCTAACGCGAGAGTTGACCGCCTGGGAAGTACGGTCGCAAGACTAAAACTCAAAGGAATTGACGGGGACCCGCACAAGCGGTGGATGATGTGGATTAATTCGATGCAACGCGAAAAACCTTACCTACCCTTGACATGGCAGACAACTCCTTGTAATGAGGAGAGCTCGCAAGAGGATCTGCACACAGGTGCTGCATGGCTGTCGTCAGCTCGTGTCGTGAGATGTTGGGTTAAGTCCCGCAACGAGCGCAACCCTTGTCACTAGTTGCTACGAAAGGGCACTCTAAGTGAGACTGCCGGTGACAAACCGGAGGAAGGTGGGGATGACGTCAAGTCCTCATGGCCCTTATGGGTAGGGCTTCACACGTCATACAATGGTCGGAACAGAGGGCAGCGAAGCCGAGAGGCGGAGCAAATYCCAAAAAACCGATCGTAGTCSGATTGCAGTCTGCAACTCGACTGCATGAAGTCGGAATCGCTAGTAATCGCGGATCAGCATGCCGCGGTGAATACGTTCCCGGTCTTGTACACACCGCCCGTCAAACAATGGGAGTGGTGTTTACCAGAAGTCGTTAGCCTAACCGCAAGGAGGGCGGCGACCACGGTGAGCACCGTGACTAATGTTAAGTCGTAACAAGGTAGCCGTACCGGAAGGTGCGGCTGGATCCTCCTTTCAAAAGTTTTTTGCTTGAAGATTTGAGTGTCCGCTCTTATCGGCAGTCGATAGATTGAAGAGAGAATAGGCCAAAGGCTAAGGGTCTTTAGCTCAGCTGGTTAGAGCACCGTCTTGATAAGGCGGGGTCGATGGTTCAAGTCCATCAAGACCCACCAAAGTCTTTCATGGGGGTATAGCTCAGCTGGGAGAGCACCTGCTTTGCAAGCAGGGGGTCAACRGTTCGATCCGTTTACCTCCACCAGATTACTGAAATAAGTGCTTAGTGAGTATTTATTTCAGGCAATTTAGAAACCTGAAAATTTTTTTTTTAAAAAATCGGAAATTTGAATCAATTGAAAACGTTTATGGAAACGTAAACGGGTAAAGATTGACGAGTYATTAACAAAGCAAAACTTTGTGAGAAACTTGAAAGCGAAGACATAAACAGCGTAATGGGACAGCGTTTAGCGGTTGCTTGTAACGCTGCCGGGCAATAGGCGGCAGGGTTATAGGATCAAGCGACTAAGTGCATGTGGTGGATGCCTTAGCGATCACAGGCGAAAGAAAGACGTGGCAGCCTGCGAAAAGCTGCGGGGAGCTGGCAAACGAGCATAGATCGCAGATATCTGAATGGGGAACCCAGCCCGATAAGGGTTATCGCATGATGAATCCATAGTCATGCGAGGCGAACGAGGAGAACTGAAACATCTAAAGTATCCTCAGGAAAAGAAATCAACCGAGATTCCGGCAGTAGCGGCGAGCGAACCCGRGARCCTAGTTTTGATAGTTTGGATAATAGAAGAATGAGATGGAAAGCTCAGCCAAAGAAGGTGATAGCCCTGTATTTAAAATTGTCCGAATGGTACTAAGGAAACGAGTAAGGCAGGACACGAGGAATCCTGTCTGAAGAAGGGGGACCATCCTCAAGGCTAAATACAGTGATCGACCGATAGTGAACCAGTACCGTGAGGGAAAGGGAAAAAAACCCCGGGGGGTGAAATAGAACCTGAAACCGCATGCATACAAACAGTAGGAGCCTTTTTTGAGGTGACTGCGTACCTTTGCATAATGGGTCAGCGACTTACATTCTGTGGCAAGGTTAACCARATAGGAAGCCGAAGCGAAAGCAGTTCGAATAGAGCGAAGAGTCGCAGGTGTAGACCCGAAACCAGATGAGCTATCCATGGCCAGGTTGAAGGTGTGGTAACACACACTGGAGGACCGAACCGACTAGTGTTGCAAAATTAGCGGATGAGCTGTGGATAGGGGTGAAAGGCCAAACAAATCTGGAGATAGCTGGTTCTCCCGAAAACTATTGAGGATAGTGGGTAGAGCACCGTTTTTGGCTAGGGGACATGTAGTCTTACAAACCAAGGCAAACTCCGAATACCTGGAAGCTATACACGGGAGACAGAGCACCGGGTGCTAACGTCCGGACTCAAAGARAAAACAGACCGCCGGCTAGGTCCCCAATAGCAGCTAAGTGGAAAACAGAAGTGGAAGGCCGTGACAACCAGGAAGTTGGCTTAGAAGCAGCCATCCTTCAAAGAAAGCGTAATAGCTCACTGGTCGAGTCTTTCTGCGCGGAAGATGTAACGGGGCTAAGCTGCTAACCGAAGCCGCGGATGCATGGATTAATCCATGCGTGGTAGGGGAGCGTTCTGTAAGTCTGAGAAGGCGGATTGAAGTCTGCTGGAGATATCAGAAGTGCGAATGCTGACATGAGTAACGTTAAAGAGGGTGAAAAGCCCTCTCGCCGCAAAACCCAAGGTTTCCTGCTCTACGTTCATCGGAGCAGGGTGAGTCRACTAAGGTCAGGCCGAAAGGCGTAGCCGATGGGAACCAGGTTAATATTCCCGGACCGCTGTCAGATGCGAAGGGGTGACGGAGGCTAAGGTCATCCAGTGTTGGACTACTCGGTACTTAGACGAGGACGGTTGGCAGGCAAATCCGCCAACGGATCGTCCGGATCGGAAGTGGTGCCCCCGGGGGGAAGTGATTGAAAACYTTCCAGGAAAAAACCTCTAAGCTACAGTCTGACAGGACCGTACCGCAAACCGACACTGGTGGGCCGAGCTGAATATGCTCAGGCGCTTGAGAGAACTCGGGAGAAGGAACTCGGCAAATTGACACCGTAACTTCGGAAGAAGGTGTGCCGCAGTAGCGTGAGAGGAGAAACACCTTGAGCGTGAGACGGTCTCAGAGAATAGGTGGCTGCGACTGTTTATTAAAACACAGCACTCTGCAAAGTCGAAAGACGACGTATAGGGTGTGATGCCTGCCAGTGCTGGAAGATTAATTGATGGGCTGAGAGGCCTTGATCGAAGTCCCAGTAAACGGCGGCCGTAACTATAACGGTCCTAAGGTAGCGAAAATTCCTTGTCGGGTAAGTTCCGACCTGCACGAATGGCATAACGATGGCCACACTGTCTCCCCCGAGACTCAGTGAAGTTGAAGTGTTTGTGATGATGCAATCTACCCGCGGCTAGACGGAAAGACCCCATGAACCTTTACTGCAGCTTTGCATTGAACTGTGAACCGATTTGTGTAGGATAGGTGGGAGACAGAGAAGAGGAGCGCCAGCTCCTCTGGAGTCAACAATGAAATACCACCCTGAT |
| Bacteria | *Uncultured Turicimonas sp.* | MN135769 | CTAAGAATAAGCACCGGCTAACTACGTGCCAGCAGCCGCGGTAATACGTAGGGGTGCGAGCGTTAATCGGAATTACTGGGCGTAAAGGGTGTGCAGGCGGTTTTGCAAGATGGATGTGAAAGCCCCGGGCTTAACCTGGGAAAACCATACATGACTGCAAGACTAGAGTGCGTCAGAGGGGGGGGAATTCCAAGTGTAGCAGTGAAATGCGTAGATATTTGGAAGAACACCGATGGCGAAGGCAGCCCCTGGGACGCAACTGACGCTCATACACAAAGCGTGGGGGGGCAAACAGGTTAGATACCCTGGTAGTCCACGCCTAAACGATGTCAACTAGTTGTTGGGAGGTAAAACTTTCAGTAACGCAGCTAACGCGAAAGTTGACCGCCTGGGAAGTACGGTCGCAAGACTAAAACTCAAAAGGAATTGACGGGGACCCGCACAAGCGGTGGATGATGTGGATTAATTCGATGCAACGCGAAAAACCTTACCTACCCTTGACATGGCAGACAACTCCTTGTAATGAGGGGGGGCTCGCAAGAGGATCTGCACACAGGTGCTGCATGGCTGTCGTCAGCTCGTGTCGTGAGATGTTGGGTTTAAGTCCCGCAACGAGCGCAACCCTTGTCACTAGTTGCTACGAAAGGGCACTCTAGGTGAGACTGCCGGTGACAAACCGGAGGAAGGTGGGGATGACGTCAAGTCCTCATGGCCCTATGGGTWGGGGCTTCACACGTCATACAATGGGTCGGAACAGAGGGCAGCAAAGCCGAGAGGCGGAGCAAATCCCAAAAACCGATCGTAGTCCGATTGCAGTCTGCAACTCGACTGCATGAAGTCGGAATCGCTAGTAATCGCGGATCAGCATGCCGCGGTGAATACGTTCCCGGGTCTTGTACACACCGCCCGTCAAACAATGGGGTGGTGTTTACCAGAAGTCGTTAGCCTAACCGCAAGGAGGGCAGCGACCACGGTGAAACACCGTGACTAATGTTAAGTCGTAACAAGGTAGCCGTACCGGAAGGTGCGGCTGGATCCTCTCCTTTCAAGGTTTTTGCTTGAAGATTTGAGTGTCCGCTCTTATCGGCAGTCGATAGATTGAAGAAGATAGTTCTTTAAAAAAGGAAATTTGAATCAATTGAAAACGTTTATGGAAACGTAAACGGGAAAAGATTGACGAGTCATAAACAAAGCAAAACTTTGTGAGAAACTTGAAAGCGAAGACATAAACAGCGTAATGGGAACAGCGTTTAGCGGTTGCTTGTAACGCTGCCGGGCAATAGGCGGCAGGGTTATAGGATCAAGCGACTAAGTGCATGTGGTGGATGCC  TTAGCGATCACAGGGSAAAAAAAGACGTGGCAGCCCTGCGAAAAGCTGCGGGGAGCTGGCAAACGAGCATAGATCCGCAGATATCTGAATGGGGGAACCCAACCCGATAGGGTTATCGCATGATGAATCCATAGTCATGCGAAGGCGAACGGGGAGAACTGAAACATCTAAGTATCCTCAGGAAAGAAATCAACCGAGATTCCGGCAGTAGCGGCGAGCAGACCGGGGGAACCCTAGTTTTTGATAGTTTGGATGATAGAAAAATGAGATGGAAAGCTCAGCCAGAGAAGGTGATAGCCCTGTATTTAAAATTGTCCAAATGGTACTAAGGAAACGAGAAGTAAGGCAGGACACGGAAWTCTGTCTCARAAGRGGGGACCATCCTCAAGGCTAAATACTAGTGATCGACCGATAGTGAACCAGTACCGTGAGGAAAGGGGAAAAAAMCCCGGGGGGAGTGAAATAGAACCTGAAACCGCATGCATACAAACAGTAGGAGCCTTTTTTTGGGGGTGACTGCGTACCTTTGCATAATGGGTCAGCGACTTACATTCTGTGGCGGGTTAACCGAATAGGRAGCCAAGCGAAAGCAGTTCGAATAGAGCGAAAAGTCGCAGGGTGTAGACCCGAAACCAGATGAGCTATCCATGGCCAGGTTGAAGGTGTGGTAACACACACTGGAGGACCGAACCGACTAGTGTTGCAAAATTAGCGGATGAGCTGTGGATGGGGTGAAAGGCCAAACAAATCTGGAGATAGCTGGTTCTCCCGAAAACTATTGAGGTAGTGCCTCGTGTGAAGGCTCCAGGGGTAGAGCACTGTTTTGGCTAGGGGGACATGTAGTCTTACCAAACCAAGGCAAACTCCGAATACCTGGAAGCTATACACGGGAGACAGAGCACCAGGTGCTAACGTCCGGACTCAAAGGAAAAAAACCCAGACCCCRGCTAGGGTCCCCAATAGCAGCTAAGTGGAAAACA  GAAGTGGAAGGCCGTGACAACCAGGAAGTTGGCTTAGAAGCAGCCATCCTTCAAAAAAGCGTAATAGCTCACTGGTCGAGTCTTTCTGCGCGGAAGATGTAACGGGGCTAAGCTGCTAACCGAAGCCGCGGATGCATGGAAAAATCCATGCGTGGTGAGAACGTTCTGTAAGTCTGAGAGGCAGATTGAGAAGTCTGCTGGAGATATCAGAAGTGCGAATGCTGACATGAGTAACGTTAAAGAGGGTGAAAACCCTCTCGCCGCAAGCCCAAGGTTTCCTGCTCTACGTTCATCRGACAGGGTGGAGTCRCCCCTAAGGTCAGGCCGAAGGCGTAGCCGATGGGAACCRGGTTAATATTCCCGGACCGCTGTCAGATGCGAAGGGGTGACGGAGAGGCTAAGGTCATCCGAGTGTTGGACTACTCGGTACTTAGACGAGGAYGGTTGGCRRGCARWYMYGSMARCGGATCATCCGGATCGGAAGTGGTGCTCCCACGGGAGCAGTGATTGAAACYCTTCCAGGAAAAAACCTCTAAGCTACAGTTTGASAGGACCGTACCGCAAACCGACACTGGTGGGCGAGCTGAATATGCTCAGGCGCTTGAGAGAACTCGGGAGAAGGAACTCGGCAAATTGACACCGTAACTTCGGAAGAAGGTGTGCCGCAGTAGCGTGAGAGGAGAGACACCTTGAGCGTGAGACGGTCTCAGAGAATAGGTGGCTGCGACTGTTTATTAAAACACAGCACTCTGCAAAGTCGAAAGACGACGTATAGGGTGTGATGCCTGCCCGGTGCTGGAAGATTAATTGATGGGCTGAGAGGCCTTGATCGAAGTCCCAGTAAACGGCGGCCGTAACTATAACGGTCCTAGGTRGGAAATTCCTTGTCGGGTAAGTTCCGACCTGCACGAATGGCATAACGATGGCCACACTGTCTCCTCCCCGAGACTCAGTGAAGTTGAAGTGTTTGTGATGATGCAATCTACCGCGGCTAGACGGGAAAGACCCCATGAACCTTTACTGCAGCTTTGCATTGAACTGTGAACCGATTTGTGTAGGATAGGTGGGAGACAGAGAAAAAGAGCGCCAGCTCCTCTGGAGTCAACTGAAATACCACCCT |

**Table S5.** **List of species detected through the Visual Encounter Record.** **It is specified if they were recorded in ponds or river transects (RT) and the year of sampling that the species were observed.**

|  | Families | Species | Records | Sites | Year |
| --- | --- | --- | --- | --- | --- |
| 1 | Leptodactylidae | *Adenomera andreae* | 1 | RT | 2020 |
| 2 | Aromobatidae | *Allobates aff. Insperatus* | 1 | Pond | 2020 |
| 3 | Hylidae | *Boana geographica* | 10 | Pond | 2020-2021 |
| 4 | Hylidae | *Boana lanciforme* | 6 | Pond | 2020-2021 |
| 5 | Hylidae | *Boana punctata* | 38 | Pond | 2020-2021 |
| 6 | Centrolenidae | *Cochranella resplendes* | 1 | RT | 2020 |
| 7 | Hylidae | *Dendropsophus bifurcus* | 36 | Pond | 2020-2021 |
| 8 | Hylidae | *Dendropsophus parviceps* | 12 | Pond | 2020 |
| 9 | Hylidae | *Dendropsophus reticulatus* | 21 | Pond | 2020-2021 |
| 10 | Hylidae | *Dendropsophus rhodopeplus* | 15 | Pond | 2020-2021 |
| 11 | Hylidae | *Dendropsophus sarayacuensis* | 11 | Pond | 2020-2021 |
| 12 | Leptodactylidae | *Leptodactylus wagneri* | 9 | Pond and RT | 2020-2021 |
| 13 | Craugastoridae | *Oreobates quixensis* | 2 | RT | 2020 |
| 14 | Craugastoridae | *Pristimantis malkini* | 2 | RT | 2020 |
| 15 | Craugastoridae | *Pristimantis variabilis* | 1 | RT | 2020-2021 |
| 16 | Bufonidae | *Rhaebo ecuadorensis* | 6 | RT | 2020-2021 |
| 17 | Bufonidae | *Rhinella marina* | 26 | Pond and RT | 2020-2021 |
| 18 | Hylidae | *Scinax cruentomma* | 1 | Pond | 2020 |
| 19 | Hylidae | *Scinax garbei* | 18 | Pond | 2020-2021 |
| 20 | Hylidae | *Scinax ruber* | 31 | Pond and RT | 2020-2021 |
|  |  | **Total** | 248 | Pond and RT | 2020-2021 |

**Table S6.** **Record of species detected by eDNA metabarcoding with their respective numbers of readings.** It is specified if they were recorded in ponds or river transects (RT) and the year they were detected.

|  | **Families** | **Species** | | **Reading** | **Sites** | **Year** |
| --- | --- | --- | --- | --- | --- | --- |
| 1 | Leptodactylidae | *Adenomera hylaedactyla* | 3 | | Pond | 2020 |
| 2 | Hylidae | *Boana geographica* | 786 | | Pond and RT | 2020-2021 |
| 3 | Hylidae | *Boana lanciforme* | 1304 | | Pond and RT | 2020-2021 |
| 4 | Hylidae | *Boana punctata* | 4341 | | Pond and RT | 2020-2021 |
| 5 | Hylidae | *Dendropsophus bifurcus* | 4067 | | Pond and RT | 2020-2021 |
| 6 | Hylidae | *Dendropsophus parviceps* | 1132 | | Pond and RT | 2020-2021 |
| 7 | Hylidae | *Dendropsophus reticulatus* | 2621 | | Pond and RT | 2020-2021 |
| 8 | Hylidae | *Dendropsophus rhodopeplus* | 2858 | | Pond and RT | 2020-2021 |
| 9 | Hylidae | *Dendropsophus sarayacuensis* | 1369 | | Pond and RT | 2020-2021 |
| 10 | Hylidae | *Dendropsophus triangulum* | 35 | | Pond | 2021 |
| 11 | Centrolenidae | *Hyalinobatrachium munozorum* | 432 | | Pond and RT | 2020-2021 |
| 12 | Centrolenidae | *Hylorcirtus phyllognatus* | 255 | | RT | 2020-2021 |
| 13 | Leptodactylidae | *Leptodactylus wagneri* | 2720 | | Pond and RT | 2020-2021 |
| 14 | Craugastoridae | *Oreobates quixensis* | 52 | | RT | 2020-2021 |
| 15 | Hylidae | *Osteocephalus buckleyi* | 348 | | Pond and RT | 2020 |
| 16 | Craugastoridae | *Pristimantis acerus* | 145 | | RT | 2020-2021 |
| 17 | Craugastoridae | *Pristimantis conspicillatus* | 43 | | RT | 2020-2021 |
| 18 | Craugastoridae | *Pristimantis eriphus* | 835 | | RT | 2021 |
| 19 | Craugastoridae | *Pristimantis malkini* | 782 | | Pond and RT | 2020-2021 |
| 20 | Craugastoridae | *Pristimantis mallii* | 729 | | RT | 2020-2021 |
| 21 | Craugastoridae | *Pristimantis sp.* | 486 | | Pond | 2020-2021 |
| 22 | Craugastoridae | *Pristimantis sp.aff.* INABIO 15591 | 85 | | RT | 2021 |
| 23 | Bufonidae | *Rhaebo ecuadorensis* | 2138 | | Pond and RT | 2020-2021 |
| 24 | Bufonidae | *Rhinella marina* | 292620 | | Pond and RT | 2020-2021 |
| 25 | Centrolenidae | *Rulyrana flavopunctata* | 10 | | RT | 2020 |
| 26 | Hylidae | *Scinax garbei* | 6877 | | Pond and RT | 2020-2021 |
| 27 | Hylidae | *Scinax ruber* | 47973 | | Pond and RT | 2020-2021 |
| 28 | Centrolenidae | *Teratohyla midas* | 10 | | RT | 2021 |
|  |  | ***Total*** | **375056** | | Pond and RT | 2020-2021 |

**Table S7.** Data used for the creation of the Sankey diagram

| **Source (Start)** | **Target (Arrival)** | **Value (Connection thickness)** |
| --- | --- | --- |
| **First node: Sampling Sites-Families** | | |
| ChP1 | Bufonidae | 0.8 |
| ChP1 | Centrolenidae | 1 |
| ChP1 | Strabomantidae | 1.04 |
| ChP1 | Hylidae | 3.91 |
| ChP1 | Leptodactylidae | 0.8 |
| ChP2 | Bufonidae | 0.4 |
| ChP2 | Strabomantidae | 1.04 |
| ChP2 | Hylidae | 5.4 |
| ChP2 | Leptodactylidae | 0.8 |
| ChP3 | Aromobatidae | 1 |
| ChP3 | Bufonidae | 0.4 |
| ChP3 | Strabomantidae | 0.52 |
| ChP3 | Hylidae | 3.9 |
| ChP3 | Leptodactylidae | 0.4 |
| TrP1 | Bufonidae | 0.8 |
| TrP1 | Strabomantidae | 2.6 |
| TrP1 | Hylidae | 2.86 |
| TrP1 | Leptodactylidae | 1.2 |
| TrP2 | Bufonidae | 0.8 |
| TrP2 | Centrolenidae | 1.04 |
| TrP2 | Strabomantidae | 3.13 |
| TrP2 | Hylidae | 1.98 |
| TrP2 | Leptodactylidae | 0.4 |
| TrP3 | Bufonidae | 0.8 |
| TrP3 | Centrolenidae | 1 |
| TrP3 | Strabomantidae | 3.65 |
| TrP3 | Hylidae | 3.96 |
| TrP3 | Leptodactylidae | 0.4 |
| **Second node: Families – Methods** | | |
| Bufonidae | VES | 2 |
| Bufonidae | eDNA | 2 |
| Centrolenidae | VES | 1 |
| Centrolenidae | eDNA | 3 |
| Strabomantidae | VES | 3 |
| Strabomantidae | eDNA | 9 |
| Hylidae | VES | 11 |
| Hylidae | eDNA | 13 |
| Leptodactylidae | VES | 2 |
| Leptodactylidae | eDNA | 2 |
| Aromobatidae | VES | 1 |
| **Third node: Methods-Species** | | |
| VES | *A.andreae* | 1 |
| VES | *A.insperatus* | 1 |
| VES | *C.resplendens* | 1 |
| VES | *P.variabilis* | 1 |
| VES | *S.cruentomma* | 1 |
| VES | *Especies en común* | 15 |
| eDNA | *Especies en común* | 15 |
| eDNA | *A.hylaedactyla* | 1 |
| eDNA | *D.triangulum* | 1 |
| eDNA | *H.munozorum* | 1 |
| eDNA | *H.phyllognathus* | 1 |
| eDNA | *O.buckleyi* | 1 |
| eDNA | *P.acerus* | 1 |
| eDNA | *P.conspicillatus* | 1 |
| eDNA | *P.eriphus* | 1 |
| eDNA | *P.mallii* | 1 |
| eDNA | *Pristimantis sp.* | 1 |
| eDNA | *D.triangulum* | 1 |
| eDNA | *Pristimantis sp. aff. INABIO15591* | 1 |
| eDNA | *R.flavopunctata* | 1 |
| eDNA | *T.midas* | 1 |
| **Species in common** | | |
| 1 | *Boana geographica* | 2 |
| 2 | *Boana lanciforme* | 2 |
| 3 | *Boana punctata* | 2 |
| 4 | *Dendropsophus bifurcus* | 2 |
| 5 | *Dendropsophus parviceps* | 2 |
| 6 | *Dendropsophus reticulatus* | 2 |
| 7 | *Dendropsophus rhodopeplus* | 2 |
| 5 | *Dendropsophus sarayacuensis* | 2 |
| 9 | *Leptodactylus wagneri* | 2 |
| 10 | *Oreobates quixensis* | 2 |
| 11 | *Pristimantis malkini* | 2 |
| 12 | *Rhaebo ecuadorensis* | 2 |
| 13 | *Rhinella marina* | 2 |
| 14 | *Scinax garbei* | 2 |
| 15 | *Scinax ruber* | 2 |

**Supplementary Figures**

1.5 k pb>

300 pb>

100 pb>

200 pb>

50 pb>


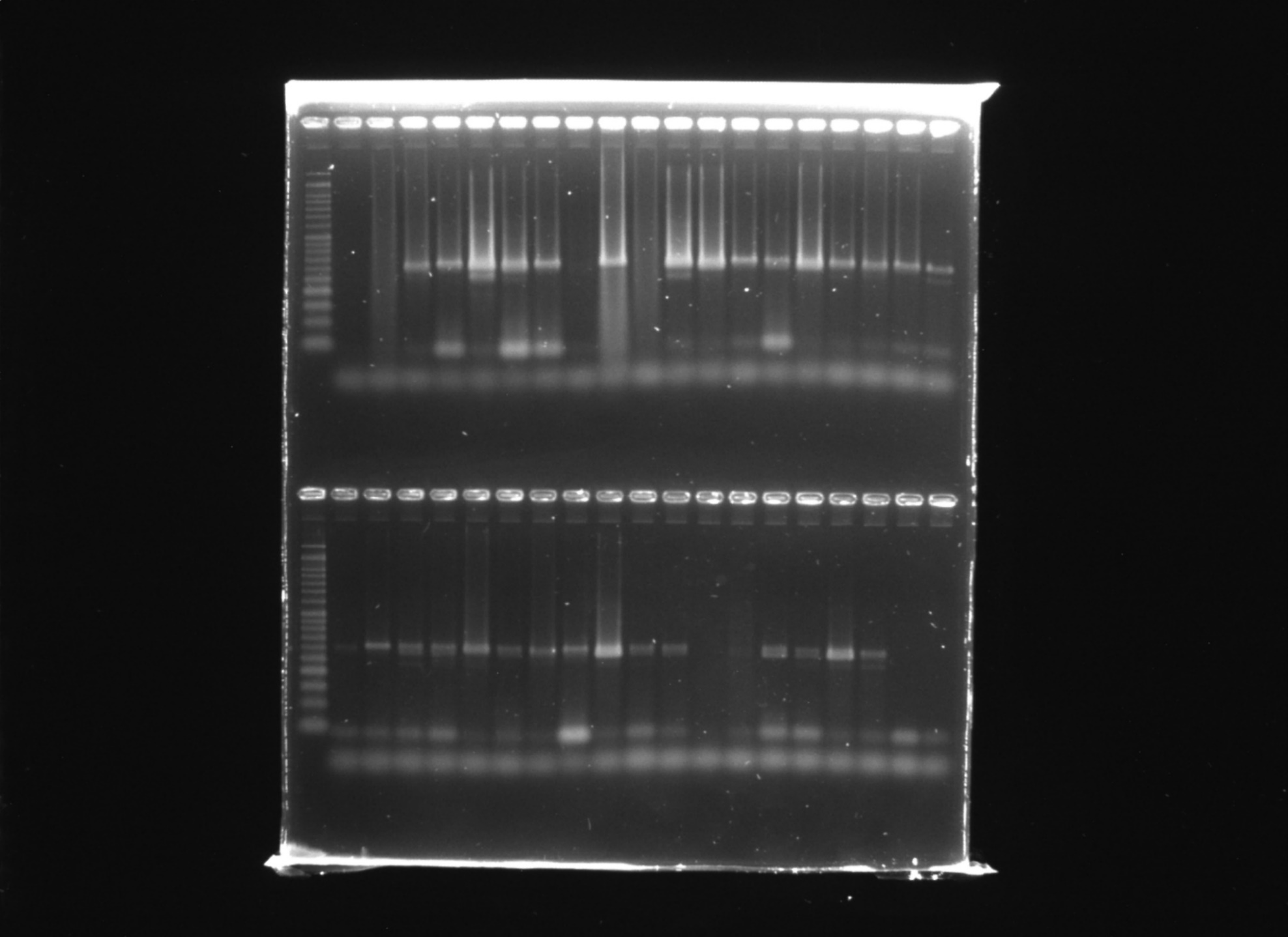


1.5k pb>

1k pb>

700 pb>

500 pb>

400 pb>

50 pb>

100 pb>

200 pb>

300 pb>

400 pb>

500 pb>

1k pb>

L

1

2

3

5

4

6

7

8

9

10

12

11

13

14

15

16

17

18

19

L

20

21

22

24

23

25

26

27

28

29

31

30

32

33

34

35

36

NTC

NRC

**Amplified samples 2020**

**Lane L:** Ladder; **Lane 1:** EchP3

**Lane 2:** EChP2; **Lane 3:** EChP1

**Lane 4:** ETrP3; **Lane 5:** ETrP2

**Lane 6:** ETrP1; **Lane 7:** FchP3

**Lane 8:** FChP2; **Lane 9:** FChP1

**Lane 10:** FTrP3; **Lane 11:** FTrP2

**Lane 12:** FTrP1; **Lane 13:** MchP3

**Lane 14:** MChP2; **Lane 15:** MChP1

**Lane 16:** MTrP3; **Lane 17:** MTrP2 **Lane 18:** MTrP1; **Lane 19:** FChP3

**Amplified samples 2021**

**Lane L:** Ladder; **Lane 20:** FChP2

**Lane 21:** FChP1; **Lane 22:** FTrP3

**Lane 23:** FTrP2; **Lane 24:** FTrP1

**Lane 25:** MchP3; **Lane 26:** MChP2

**Lane 27:** MChP1; **Lane 28:** MTrP3 **Lane 29:** MTrP2; **Lane 30:** MTrP1; **Lane 31:** AChP3; **Lane 32:** AChP2

**Lane 33:** AChP1; **Lane 34:** ATrP3

**Lane 35:** ATrP2; **Lane 36:** ATrP1.

**Lane NTC:** Negative Control 1

**Lane NRC:** Negative Control 2


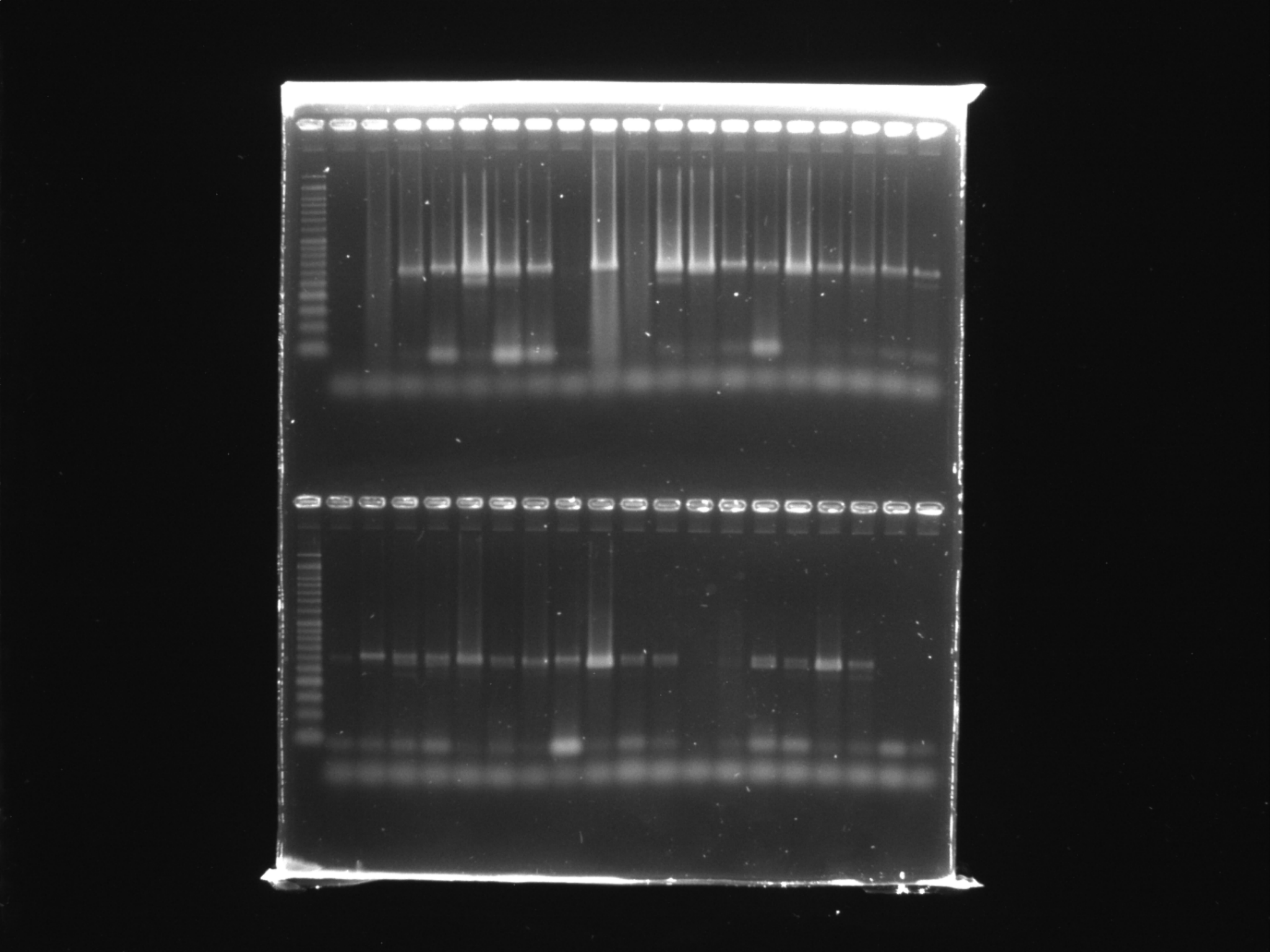

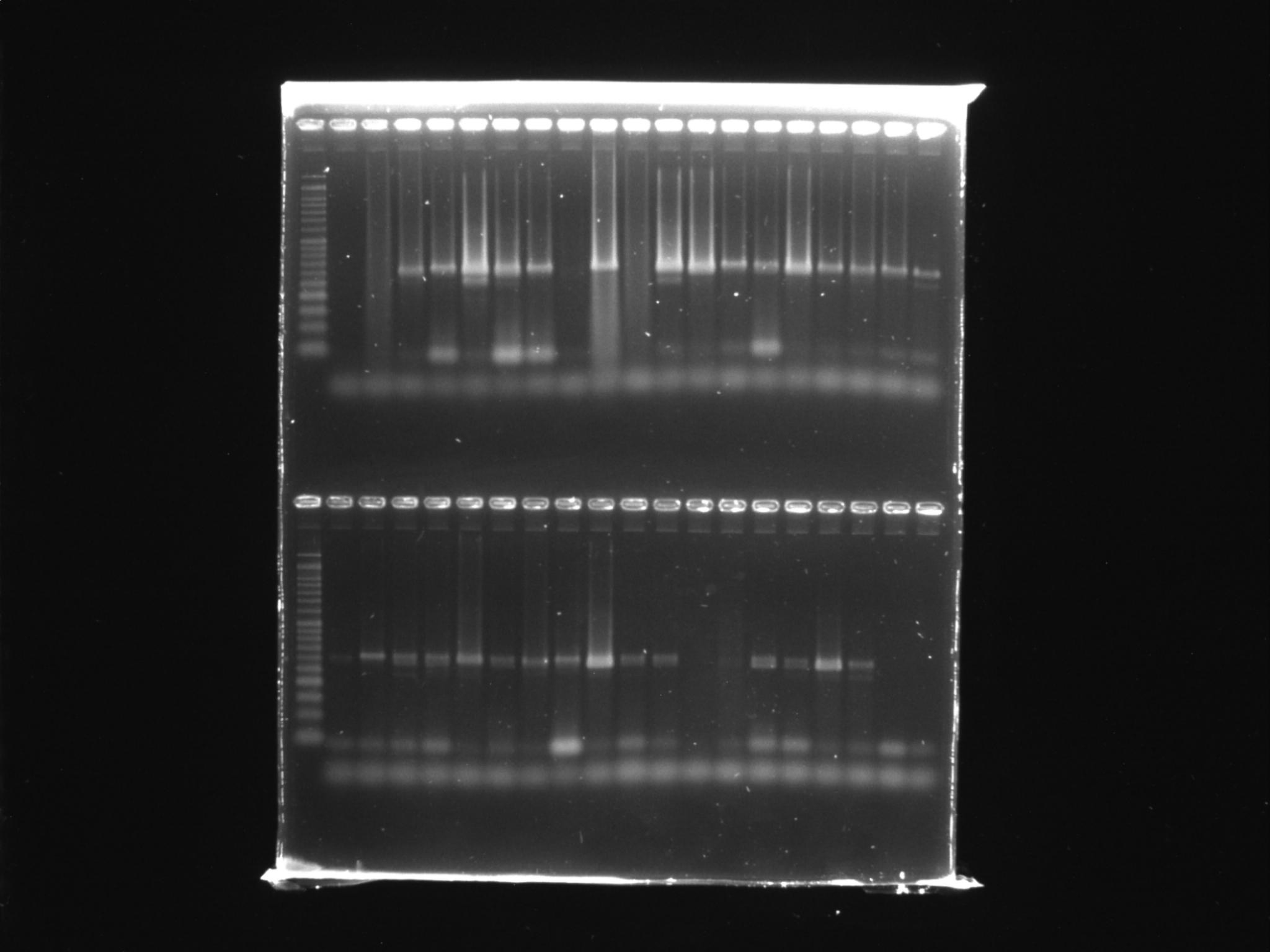


**Figure. S1.** **Electrophoretic gel 2% (m/v) of environmental samples.** Lane L. 100 bp molecular weight marker (BioGENA). Lanes 1-36: eDNA PCR products with Vert 16S eDNA F1/R1 primers with adapters for ONT approx. 300 bp. Lane NTC: negative transport control. Lane NRC. Negative reaction control.

**Figure. S2.** **Electrophoretic gel 2% (m/v) negative transport control.**  Lane L. 100 bp molecular weight marker (BioGENA). Lanes 1-12: PCR products of negative transport controls from the six months of sampling with Vert 16S eDNA F1/R1 primers with adapters for ONT approx. 300 bp. Lane 13: negative reaction control.

1.5 k pb>

1k pb>

500 pb>


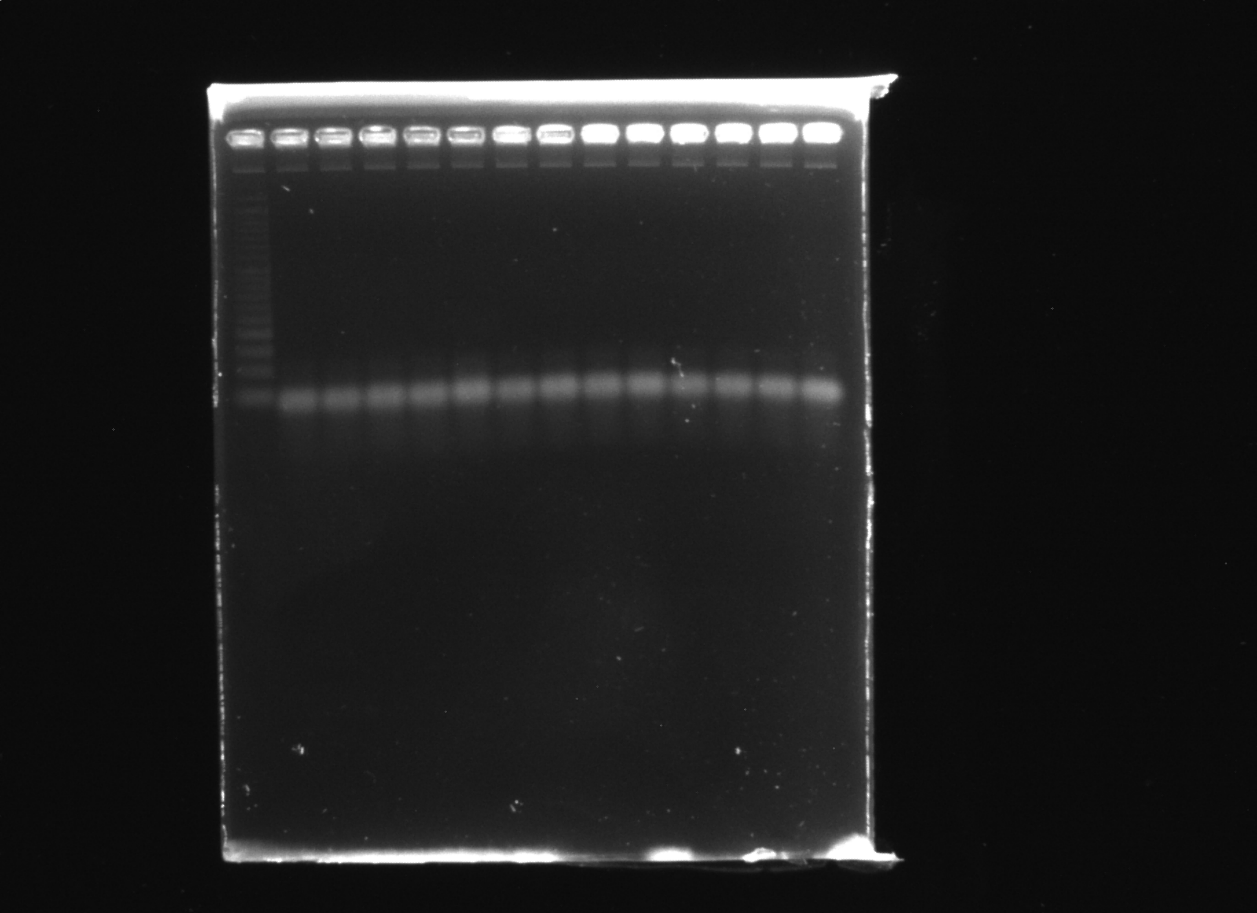


50 pb>

100 pb>

200 pb>

300 pb>

600 pb>

L

1

2

3

4

5

7

6

8

9

10

11

12

C-

**Amplified samples**

**Lane L:** Ladder.

**Lane 1:** Negative control 1, January 2020

**Lane 2:** Negative control 2, January 2020

**Lane 3:** Negative control 1, February 2020

**Lane 4:** Negative control 2, February 2020

**Lane 5:** Negative control 1, March 2020

**Lane 6:** Negative control 2, March 2020

**Lane 7:** Negative control 1, February 2021

**Lane 8:** Negative control 2, February 2021

**Lane 9:** Negative control 1, March 2021

**Lane 10:** Negative control 2, March 2021

**Lane 11:** Negative control 1, April 2021

**Lane 12:** Negative control 2, April 2021

**Lane 13:** Negative reaction control
